# Repeated out-of-Africa expansions of *Helicobacter pylori* driven by replacement of deleterious mutations

**DOI:** 10.1038/s41467-022-34475-3

**Published:** 2022-11-11

**Authors:** Harry A. Thorpe, Elise Tourrette, Koji Yahara, Filipa F. Vale, Siqi Liu, Mónica Oleastro, Teresa Alarcon, Tsachi-Tsadok Perets, Saeid Latifi-Navid, Yoshio Yamaoka, Beatriz Martinez-Gonzalez, Ioannis Karayiannis, Timokratis Karamitros, Dionyssios N. Sgouras, Wael Elamin, Ben Pascoe, Samuel K. Sheppard, Jukka Ronkainen, Pertti Aro, Lars Engstrand, Lars Agreus, Sebastian Suerbaum, Kaisa Thorell, Daniel Falush

**Affiliations:** 1 Department of Biostatistics, University of Oslo, Oslo, Norway; 2 CAS Key Laboratory of Molecular Virology and Immunology, Institut Pasteur of Shanghai, Chinese Academy of Sciences, Shanghai, China; 3 Antimicrobial Resistance Research Center, National Institute of Infectious Diseases, Tokyo, Japan; 4 Pathogen Genome Bioinformatics and Computational Biology, Research Institute for Medicines (iMed.ULisboa), Faculty of Pharmacy, Universidade de Lisboa, Lisbon, Portugal; 5 University of Chinese Academy of Sciences, Beijing, China; 6 National Reference Laboratory for Gastrointestinal Infections, Department of Infectious Diseases, National Institute of Health Dr Ricardo Jorge, Lisbon, Portugal; 7 Department of Microbiology, Hospital Universitario La Princesa, Instituto de Investigación Sanitaria Princesa, Madrid, Spain; 8 Gastroenterology Laboratory, Rabin Medical Center, Petah Tikva, Israel; 9 Department of Digital Medical Technologies, Holon Institute of Technology, Holon, Israel; 10 Department of Biology, Faculty of Sciences, University of Mohaghegh Ardabili, Ardabil, Iran; 11 Department of Environmental and Preventive Medicine, Oita University Faculty of Medicine, Yufu, Oita Japan; 12 Department of Medicine-Gastroenterology, Baylor College of Medicine, Houston, TX USA; 13 Laboratory of Medical Microbiology, Hellenic Pasteur Institute, Athens, Greece; 14 G42 Healthcare, Abu Dhabi, UAE; 15 Elrazi University, Khartoum, Sudan; 16 Department of Biology, University of Oxford, Oxford, UK; 17 Ineos Oxford Institute, Department of Biology, University of Oxford, Oxford, UK; 18 Center for Life Course Health Research, University of Oulu, Oulu, Finland; 19 Primary Health Care Center, Tornio, Finland; 20 Arokero Oy, Tornio, Finland; 21 Center for Translational Microbiome Research, Department for Microbiology, Tumor, and Cell Biology, Karolinska Institutet, Stockholm, Sweden; 22 Division of Family Medicine, Karolinska Institutet, Stockholm, Sweden; 23 Department of Medical Microbiology and Hospital Epidemiology, Max von Pettenkofer Institute, Faculty of Medicine, LMU Munich, Munich, Germany; 24 Department of Medical Microbiology and Hospital Epidemiology, Hannover Medical School, Hanover, Germany; 25 DZIF German Center for Infection Research, Hannover-Braunschweig and Munich Partner Sites, Munich, Germany; 26 Institute of Biomedicine, Department of Infectious Diseases, University of Gothenburg, Gothenburg, Sweden; 27 Department of Clinical Microbiology, Sahlgrenska University Hospital, Gothenburg, Sweden

**Keywords:** Genetic variation, Bacterial genomics

## Abstract

*Helicobacter pylori* lives in the human stomach and has a population structure resembling that of its host. However, *H. pylori* from Europe and the Middle East trace substantially more ancestry from modern African populations than the humans that carry them. Here, we use a collection of Afro-Eurasian *H. pylori* genomes to show that this African ancestry is due to at least three distinct admixture events. *H. pylori* from East Asia, which have undergone little admixture, have accumulated many more non-synonymous mutations than African strains. European and Middle Eastern bacteria have elevated African ancestry at the sites of these mutations, implying selection to remove them during admixture. Simulations show that population fitness can be restored after bottlenecks by migration and subsequent admixture of small numbers of bacteria from non-bottlenecked populations. We conclude that recent spread of African DNA has been driven by deleterious mutations accumulated during the original out-of-Africa bottleneck.

## Abstract (web summary)

*Helicobacter pylori* is a major human pathogen whose population structure is similar to that of its host. Here, the authors show that *H. pylori* has repeatedly spread out of Africa recently, replacing deleterious variants that accumulated during the original out of Africa migrations more than 50,000 years ago.

## Introduction

H*elicobacter pylori* is the dominant bacterial member of the human stomach microbiota in infected individuals and is the etiological agent in most cases of gastric cancer, gastric mucosa-associated lymphoid tissue (MALT) lymphoma, and gastroduodenal ulcer disease^1^. *H. pylori* causes chronic, decades-long infections and is often acquired within the household, limiting the rate of its diffusion through human populations in comparison with more readily transmissible pathogens^2^. Genetic variation in *H. pylori* genome sequences shows a phylogeographic pattern similar to that of its host, consistent with an inference that human and bacterial genes are often spread by the same migrations^3–6^. However, the *H. pylori* population found in Europe and other parts of Eurasia is admixed, with many strains having more than half of their DNA attributable to populations closely related to those prevalent in Africa^3,7–9^. There is evidence for several recent human migrations out of Africa^10^, but together they have only contributed a small fraction of the ancestry of non-Africans. This discrepancy in ancestry proportions between the bacteria and their hosts implies that African *H. pylori* has been spread to Eurasia by movements of people that have left weaker signals in human DNA.

To understand why African *H. pylori* have contributed extensive ancestry within parts of Eurasia, we assemble a collection of strains from Europe and the Middle East, from putative source populations in Africa, as well as from less-admixed strains in Asia. We infer a recent demographic history of the European and Middle Eastern strains that includes genetic drift, migration, and admixture from external sources. We show that there have been at least three admixture events from African source populations that have each contributed substantial ancestry. By examining the distribution of non-synonymous mutations in different populations, we conclude that there was a large and lasting increase in the frequency of segregating deleterious mutations during the out-of-Africa bottleneck associated with the initial spread of modern humans from Africa. When African *H. pylori* strains reached Eurasia due to later contact between humans, they, and the DNA they carried, had a fitness advantage and were able to spread. In the process, they reduced the mutational load in the newly admixed populations.

## Results

### Repeated African admixture into Europe and the Middle East

**Fig. 1:**
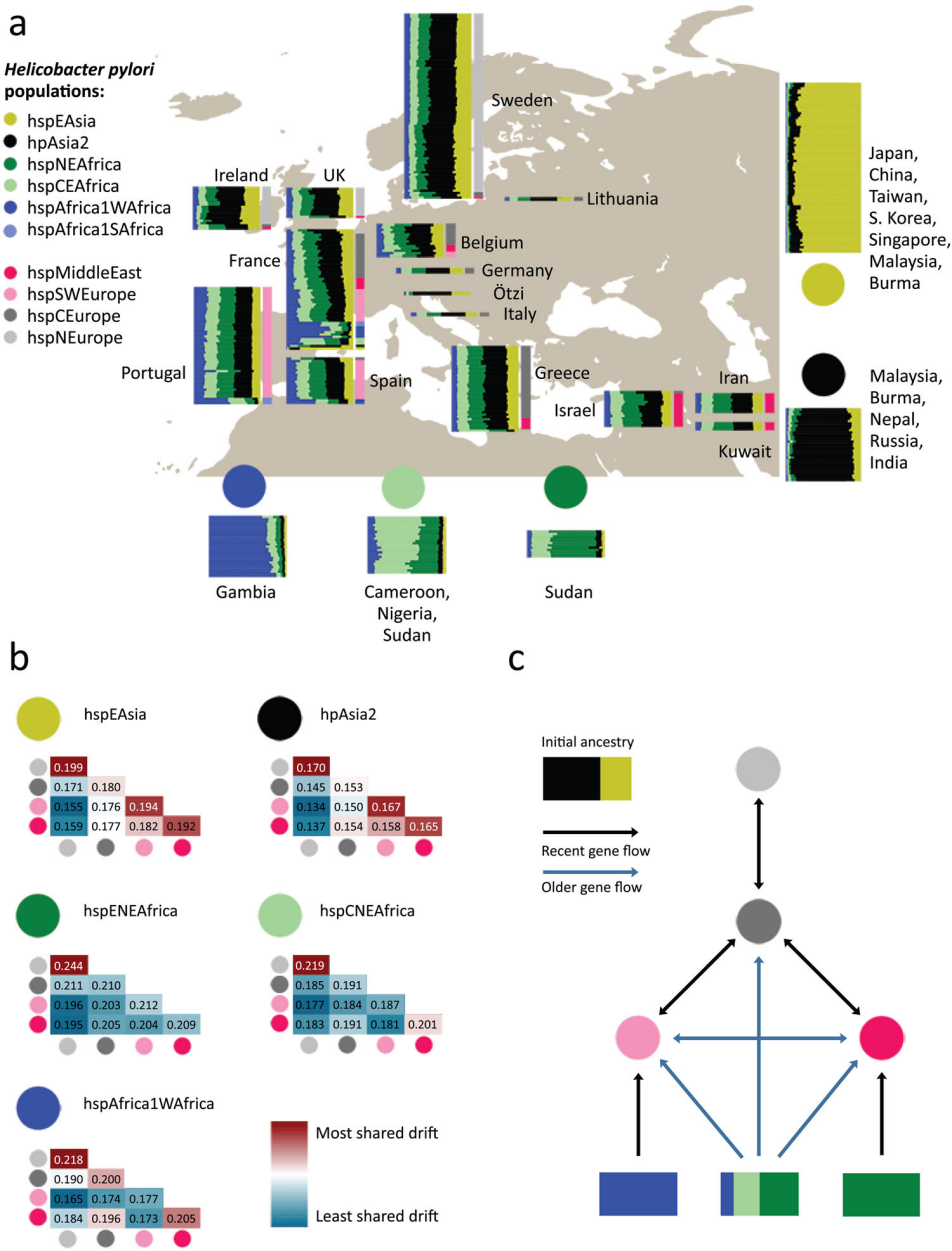
Ancestry and migration history of hpEurope isolates. **a** Painting profiles of hpEurope isolates and their putative ancestral populations from Africa and Asia showing proportion of each genome (horizontal bar) painted by each of five ancestral donor populations (circles). hpEurope isolates are grouped by country of isolation, with bars to the right indicating the *H. pylori* population each strain is assigned to. The representative isolates from each donor population are grouped by population, with countries of isolation listed for each group. **b** Genetic drift profiles for hpEurope subpopulations, shown separately for each ancestry component. **c** Schematic summarizing the migration and admixture history of the hpEurope subpopulations. Source data are provided as a Source Data file. The map in **a** is from https://commons.wikimedia.org/wiki/File:World_map_blank_gmt.png.

Based on fineSTRUCTURE clustering (Supplementary Fig. 1), we grouped European and Middle Eastern strains into four subpopulations named hspEuropeNEurope, hspEuropeCEurope, hspEuropeSWEurope and hspEuropeMiddleEast according to the locations they were most isolated from. The first Europe in the name indicates they are subpopulations of hpEurope but for brevity we omit this part of the name in the rest of the manuscript. We investigated their sources of external ancestry by performing in silico chromosome painting (Fig. 1a) using donor strains from three African populations (hspENEAfrica, hspCNEAfrica, hspAfrica1WAfrica) and two Asian populations (hpAsia2 and hspEAsia). Representative strains from these populations were selected from a larger collection on the basis that they showed little sign of recent admixture from other continents based on D-statistics (Supplementary Data 6 and 7) and as confirmed in a PCA plot (Supplementary Fig. 2).

The chromosome painting analysis supported previous findings that there is a North-South cline in the overall proportion of African ancestry and that hpAsia2 is a closer relative of the pre-admixture population than hspEAsia^3,8^. However, all isolates are painted with a substantial and largely consistent fraction of hspEAsia (with the hpAsia2:hspEAsia ratio varying from 1:1.74 for hspNEurope to 1:2.02 for hspMiddleEast), implying that hpAsia2 is not a close surrogate for the pre-admixture population.

To investigate the demographic history of admixture further, we measured genetic drift profiles separately for each ancestry component (Fig. 1b). Specifically, we compared the painting profile of pairs of admixed individuals to identify regions of the genome in which they were painted by the same donor population. For these genomic regions, we recorded if they were painted by the same specific donor strain (Methods). High rates of painting by the same donor strain indicates shared genetic drift within that ancestry component. These values are similar in interpretation to F3 values^11^, but are specific to individual ancestry components, rather than averaged across the entire genome.

Genetic drift profiles for the hpAsia2 and hspEAsia ancestry components showed a similar pattern across the four hpEurope subpopulations, as indicated by a near identical pattern of colors for these two ancestry components in Fig. 1b. The indistinguishable drift profiles provide evidence that within each hpEurope subpopulation, both components have been affected similarly by genetic drift. The simplest explanation is that these components are both being used to paint a single ancestry source that persisted in western Eurasia since the out-of-Africa bottleneck. Therefore, the data does not provide evidence for either ancient or more modern genetic contributions from the East.

In addition to the North-South cline, there is also an East-West ancestry cline in the source of African admixture (Fig. 1a), with distinct drift patterns for each African component in the four hpEurope subpopulations (Fig. 1b). Strains from hspSWEurope have the highest fractions of hspAfrica1WAfrica and this ancestry component shows low levels of drift, implying that this subpopulation has undergone recent admixture from strains closely related to those currently found in West Africa. Furthermore, there are strains from Spain, Portugal and France assigned to hspAfrica1 subpopulations and a strain in Portugal with an intermediate ancestry profile suggesting that these isolates have arrived within the last few human generations. Strains from the hspMiddleEast subpopulation have the highest fraction from hspENEAfrica and the lowest levels of drift in this component. These two populations have therefore received genetic material from Africa after the initial gene flow that has introduced ancestry across the continent. These patterns imply that there have been at least three separate admixture events involving distinct populations of African bacteria (Fig. 1c). Confirmation of these results is provided by an admixture graph estimated by Treemix^12^ (Supplementary Fig. 3). Treemix estimated nine migration events across the 11 populations in this study, three of which were from Africa into the European sub-populations. These three events corresponded closely to the three events found using chromosome painting.

The drift components also provided evidence about local migration. hspMiddleEast and hspSWEurope have high levels of shared drift in both Asian components, implying that prior to admixture, these two populations were closely related. hspNEurope has high subpopulation-specific drift in all ancestry components, showing that it has undergone recent genetic drift, while hspCEurope has almost none in any component, suggesting that it has been a hub for migration between populations.

The genome of a strain colonizing the Tirolean iceman, Ötzi, has been inferred to be a nearly pure representative of the pre-admixture population based on more limited data, which was interpreted as evidence that most of the admixture took place in the 5300 years since his death^8^. In our fineSTRUCTURE analysis, the Ötzi genome clusters with hspNEurope isolates from Ireland and Sweden with the lowest African ancestry, one of which has the same non-African ancestry proportion as the Ötzi strain in the chromosome painting. These results show that the Ötzi genome, in fact, falls within modern variation in ancestry proportions in Europe. We interpret this as evidence that substantial African ancestry had already been introduced into Europe when Ötzi lived but that ancestry proportions in particular locations have changed substantially in subsequent millennia.

Overall, the chromosome painting results show that, in addition to contemporary migrations that have introduced *H. pylori* with atypical profiles into countries such as Ireland, Portugal, France and Spain (Fig. 1a), *H. pylori* have spread out of Africa at least three times (Fig. 1c). Each of these migrations is sufficiently old that the DNA has been absorbed into the local gene pools, leading to a high degree of uniformity in ancestry profiles for most isolates in individual locations (Fig. 1a). At least one of the early admixture events was shared between the four subpopulations, spanning Europe and the Middle East, and left traces in Ötzi’s *H. pylori* genome, while later ones, labeled as recent in Fig. 1c, had foci in South Europe and the Middle East, respectively. Gene flow between the regional subpopulations has affected all ancestry components but has not been sufficient to homogenize ancestry proportions across the continent.

### Evidence for a role of deleterious mutations in the repeated expansion of bacteria from Africa

The ability of *H. pylori* of African origin to spread effectively in non-African populations on multiple independent occasions is unexpected, since the resident bacteria will have had an opportunity to adapt to local conditions. One potential explanation is that deleterious mutations accumulated in the genomes of strains carried by the early waves of modern humans that spread from Africa. Demographic bottlenecks associated with these migrations have been sufficient to leave an imprint on neutral genetic variation within the human genome, which indicates a reduction in effective population size in the ancestry of non-African humans around 50,000 years ago, followed by more recent expansion^13^. *H. pylori* populations also show evidence of low ancestral population sizes in East-Asian and native American populations, followed by population size recoveries^6^ consistent with strong genetic drift during the out-of-Africa and subsequent bottlenecks.

Population genetic theory^14^ and evidence from experimental systems^15^ has shown that demographic bottlenecks can lead to reduction in average fitness through processes such as fixation of deleterious mutations of small effect or the stochastic loss of the fittest genomes (Muller’s ratchet^16,17^). However, although the out-of-Africa bottlenecks have had measurable impact on the pattern of segregating mutations within human populations^18^, there is less evidence that individuals from non-African populations have accumulated a larger burden of non-synonymous mutations when measured relative to an outgroup^19^. *H. pylori* shows much higher rates of genetic differentiation between geographical regions^4^ than its host^13^, which most likely reflects transmission bottlenecks during spread from person to person as well as the expansion of fit clones. Consequently, demographic bottlenecks that have had modest fitness consequences for humans can potentially have more substantial effects on the bacteria they carry.

**Fig. 2:**
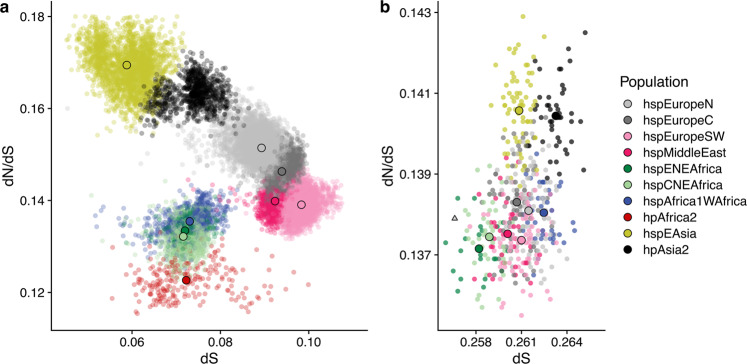
Within- and between-population divergence. **a** Within population dN/dS (*y* axis) plotted against dS (*x* axis). Small dots show pairwise distances; larger solid dots indicate population means. **b** dN/dS, calculated to the *H. acinonychis* outgroup, plotted against dS for isolates (semi opaque points) and populations (solid points), excluding hpAfrica2 isolates. The triangle indicates the genome from Ötzi. Source data are provided as a Source Data file.

Bacteria from the hspEAsia and hpAsia2 populations, which have been through the out-of-Africa bottleneck, had a higher number of deleterious mutations segregating between strains (Fig. 2a) and a higher dN/dS to the *H. acinonychis* outgroup (Figs. 2b and S4B) than do the three African populations with comparable synonymous divergence levels dS. dN/dS for the three African populations ranged between 0.137-0.138, with Asian populations having values of 0.140-0.141. This corresponded to an average of 574 extra non-synonymous mutations in the Asian populations or 1 mutation for every 3 genes. dN/dS values within populations varied between 0.123–0.133 for African populations and 0.163–0.169 for the Asian ones. This difference corresponded to an average of 1216 non-synonymous differences between pairs of strains or about 2 mutations for every 3 genes. These results show that the increase in dN/dS to the outgroup in the Asian populations reflects a larger number of segregating non-synonymous variants and thus cannot simply be attributed to fixation of mutations during the out-of-Africa bottleneck.

We confirmed the robustness of these results in two ways. Firstly, we used GRAPES^20^ to estimate the rate of non-adaptive non-synonymous substitutions based on the pattern of segregating mutations within each population. This approach can be applied with a folded-mutation spectrum, obviating the need for an outgroup. The results were highly concordant with those obtained for dN/dS values (Supplementary Fig. 5). Secondly, when an hpAfrica2 strain was used as an outgroup instead of *H. acinonychis*, similar results were obtained for dN/dS, but the range of dS values between populations was twice as large (Supplementary Fig. 6). This suggested that there have been ancient admixture events involving Africa2-like lineages and other African populations, while confirming the higher mutation load in Asian populations.

We also observed a reduction of the mutational load by admixture and selection within hpEurope subpopulations. Bacteria from hpEurope subpopulations had dN/dS values with the *H. acinonychis* outgroup that were intermediate between African and Asian populations but were lower (0.137–0.138) than would be predicted if they were random mixtures of African and Asian genomes with the proportions estimated by chromosome painting in Fig. 1a (0.139–0.140). However, this mutation deficit is not on its own compelling evidence for a direct benefit of admixture, since it might instead reflect differences in dN/dS between the population that existed in Europe prior to admixture and the Asian populations that were used as surrogates for this ancestry in this analysis.

**Fig. 3:**
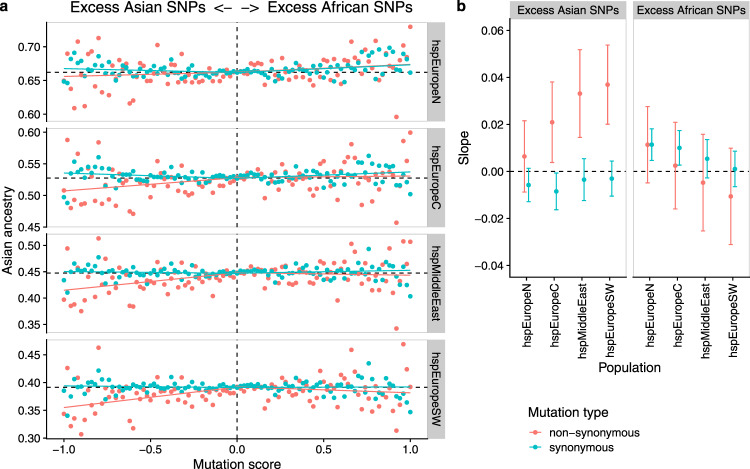
Genetic ancestry of hpEurope subpopulations as a function of mutation score. **a** Average ancestry in chromosome painting analyses plotted against mutation score (mutation frequency in African populations minus mutation frequency in Asian populations) in bins of 0.02. Regression lines were calculated separately for positive and negative mutation scores from the unbinned data. **b** Mean of the regression slopes pseudovalues with 95% confidence intervals estimated using a gene-by-gene jackknife for excess Asian and excess African mutations, respectively. For each point, the average was calculated over 840 occurrences. Source data are provided as a Source Data file.

More specific evidence that admixture has reduced the burden of deleterious non-synonymous mutations was provided by tabulating the effect of mutations that accumulated in African and Asian populations on the ancestry of admixed bacteria in Europe and the Middle East (Fig. 3). For each position in the alignment, we calculated a mutation score, which is the difference between African and Asian strains in the proportion of nucleotides that differed from *H. acinonychis* (with equal weight given to each subpopulation, see methods). We then investigated whether there was variation in overall Asian ancestry proportion (hpAsia2 + hspEAsia in the chromosome painting) associated with this score. Most sites were non-polymorphic and had a mutation score of 0.0, which was therefore used as a baseline and compared independently to positive and negative scoring sites. To allow for correlations between adjacent sites, statistical significance of the regression was assessed using a gene-by-gene jackknife (Methods).

*H. pylori* shows little evidence of codon usage bias^21^, so most synonymous mutations should be approximately neutral and can therefore be used as a control. A negative mutation score at non-synonymous sites was associated with a deficit in Asian ancestry, either in comparison with non-polymorphic sites or with synonymous sites with the same scores. The strongest regression slopes were estimated for the two Southern populations, and the weakest for hspNEurope (Fig. 3a, b). By contrast, excess mutations in African strains did not have a detectable effect on ancestry in any of the four hpEurope subpopulations, with regression slopes statistically indistinguishable from those found for synonymous positions. Thus, admixed bacteria have avoided non-synonymous mutations that accumulated in populations that have been through the out-of-Africa bottleneck, but not those that have not. We inferred that a proportion of these non-synonymous mutations are deleterious and have been selected against in the admixed population. Similar results were obtained, albeit with lower statistical confidence, when an hpAfrica2 strain was used as an outgroup instead of *H. achinonychis* (Supplementary Fig. 7A, B).

Deleterious mutations occurred throughout the genome and were all subject to genetic drift. A plot of mutation score versus ancestry at a gene-by-gene level showed considerable scatter (Supplementary Fig. 8A and Supplementary Data 8), with similar results obtained when the analysis was performed for 10 kb regions (Supplementary Fig. 8B), suggesting that the signal that we observed for mutation replacement cannot be attributed to a small number of genes. Nevertheless, it is possible that other selective forces, for example related to local adaptation, could explain some of the variation in ancestry proportion between genes.

We investigated whether specific genes were enriched for African ancestry. There was substantial variation amongst genes in the average African ancestry proportion, with strong correlations between proportions in the four hpEurope subpopulations (Supplementary Fig. 9), which is consistent with much of the ancestry resulting from a single shared admixture event. However, we observed no significant differences between COG categories in average ancestry proportion (Supplementary Fig. 10).

Genes with extreme values of average ancestry proportion were involved in diverse, often central, cellular processes. For example, low African ancestry (top ten in Supplementary Data 8, each with <27% average African ancestry) genes included, e.g., those in central energy generation (HP0145, component of the Cbb3-type terminal oxidase), stress tolerance (HP0278, *ppx* exopolyphosphatase; HP0600, *spaB* multidrug resistance), cell envelope biogenesis (HP0867, lipid A), and translation (HP1147, ribosomal proteins L19). However, the analysis did reveal overlap of low-admixture genes with genes that have highly differentiated SNPs within East Asia^22^, specifically HP0284 (*mscS-1*), encoding a mechanosensitive channel related to osmotolerance and HP0250 (*oppD*), encoding an oligopeptide permease. Since the same genes are often differentiated in different continents^22^, this overlap suggests that these genes may have been resistant to admixture due to local adaptation within Europe and the Middle East.

High-average African ancestry genes (>69%) likewise included those in central biological processes and stress tolerance, such as those of the Czc cation efflux system (HP0969-HP0970, metal ion tolerance and nickel homeostasis), purine salvage (HP0267, cytosine/adenine deaminase), and glycolysis (HP1166, glucose-6-phosphate isomerase & HP0154, enolase). Most were not directly related to host interactions, although some with low average African ancestry might affect urease activity (HP1129, *exbD* and HP0969-HP0970, *czc*). Overall, this inspection suggested that the driving force for admixture within the core genome was increased fitness, provided by replacement of stochastic deleterious mutations genome-wide, rather than single pathogenesis-related genes allowing adaptation to new host environments.

**Fig. 4:**
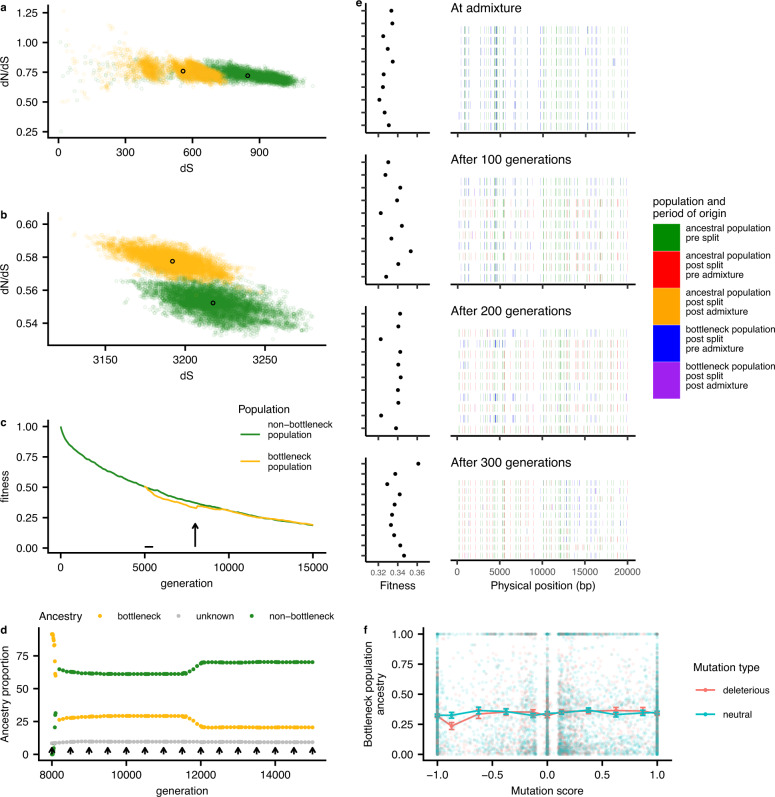
Simulations of populations under bottlenecks and admixture. **a** Within population dN/dS and **b** dN/dS calculated to the ancestor measured at generation 8000. Semi opaque points show pairwise distances; solid points indicate population means. **c** Population average fitness shown during the generations of the simulation, with bottleneck starting at generation 5000 (horizontal line) and admixture at generation 8000 (arrow). **d** Proportion of bottleneck and non-bottleneck ancestry in the bottleneck population, for the generations after the beginning of admixture. Sites with unknown ancestry are shown in gray. Each arrow corresponds to the migration of one strain. **e** Ancestry painting in a sample of genomes from the bottleneck population, in the generations subsequent to admixture. Only the first 20 kb of each genome is shown. **f** Average bottleneck population ancestry, at generation 8300 plotted against mutation score (frequency in the non-bottleneck population minus the frequency in the bottleneck population before the admixture begins). Error bars show the standard error on the mean (for each bin, from −1 to 1, the sample size is 1369, 344, 453, 639, 1264, 914, 666, 381, 241, 251, 1839). Source data are provided in the github repository https://github.com/EliseTourrette/Hpylori/tree/main/HpEurope (DOI: 10.5281/zenodo.7130003).

To test the hypothesis that selection on deleterious mutations can explain the observed patterns, we performed simulations of bacterial populations evolving with a constant input of neutral and deleterious mutations and homologous recombination of short tracts (Fig. 4 and Supplementary Figs. 11 and 12). These simulations showed that at high recombination rates, demographic bottlenecks could generate long-term increases in the number of deleterious mutations segregating in the population (Fig. 4a and Supplementary Fig. 12C, D), and in dN/dS measured relative to an outgroup (Fig. 4b and Supplementary Fig. 12E, F). These patterns qualitatively match those seen in the data (Fig. 2a, b).

In our simulations, the average fitness of the bottlenecked population underwent the largest decrease at intermediate recombination rates (Fig. 4c and Supplementary Fig. 12A, B) and in this simulation, the bottlenecked population was susceptible to invasion from strains from the non-bottlenecked population. Once an invading strain became established in the non-bottlenecked population, the frequency of migrant DNA increased in an almost stepwise fashion leading to the generation of highly mosaic genomes (Fig. 4d, e). As migrant DNA spread through the population, mutations from the bottlenecked population that were deleterious fell faster in frequency than mutations at neutral sites (Fig. 4f) reproducing the dependence observed in the data (Fig. 3a). This showed that the interplay between recombination and selection can explain the reduced mutational burden of the admixed populations.

Our simulations are consistent with results obtained for eukaryotic systems, which have shown that population bottlenecks can increase mutational load^23^ and that gene flow from populations with a higher effective population size to those with a smaller one decreases the genetic load of the smaller population^24^. This effect is particularly strong in regions of higher recombination, for which introgressed neutral/beneficial mutations will persist due to their uncoupling with the introgressed deleterious mutations. However, our simulations show that for bacterial populations the decrease of the genetic load due to admixture is only valid for intermediate recombination levels. For higher recombination rates, admixture has no effect on the fitness of the smaller population, since selection is already effective in removing deleterious mutations.

## Discussion

Deleterious mutations provide a compelling explanation for the repeated spread of African *H. pylori* into other continents, as shown by the good qualitative match between our simulation results and the pattern of diversity within contemporary *H. pylori* populations. First, we find elevated dN/dS values in Asian populations, consistent with a higher load of deleterious mutations accumulating during the out-of-Africa bottleneck. Second, our simulations show that rare migrants from non-bottlenecked populations can spread and generate highly mosaic genomes, as observed in Europe and the Middle East. Third, we show that ancestry from these invading lineages is higher in regions of the genome where non-synonymous mutations are segregating within the bottlenecked populations, implying that selection has acted to purge these mutations during admixture.

Deleterious mutations have been shown to be important during hybridization in several eukaryotic systems, including swordfish^25^, trees^26^ and Neanderthal introgression into modern humans^27^. In these systems, recombination rate variation has been shown to be crucial in determining the rate of introgression that has taken place in different regions of the genomes. To our knowledge, this is the first demonstration of a load effect in bacteria. *H. pylori* is known to recombine at an extraordinary rate^28–30^ and this may explain why a clear signal is observable in this species.

Many of the details concerning the spread of African ancestry remain to be elucidated. The initial admixture event(s) left traces in the *H. pylori* genome of the Tirolian Iceman, Ötzi, which has an ancestry profile like that found in modern day samples in Ireland and Sweden but has less African ancestry than in modern genomes from Southern Europe. Therefore, the first admixture event occurred more than 5300 years ago but the average level of African ancestry has increased substantially in the last few millennia.

*H. pylori* of different origins can mix together and recombine within extended families^31^. Over time, extensive ongoing contacts between populations within Europe would be expected to homogenize *H. pylori* ancestry proportions both within and between locations. However, we lack quantitative information on transmission dynamics that might allow us to estimate mixture dates based on the properties of the ancestry clines we observe.

An intriguing question is why some populations appear to have been resistant to invasion by DNA from African *H. pylori*. For example, within East Asia, most strains appear to come from the hpEAsia population, with very little evidence of admixture. Lack of contact with Africans does not seem a sufficient explanation, given the large number of documented contacts between East Asians and other Eurasian populations within the last several thousand years. The high burden of *H. pylori* related gastric disease in the region is notorious and hpEAsia bacteria are known for distinctive variants at virulence associated loci including *cagA* and *vacA*^32^. It is possible that strains from this population have acquired a suite of adaptations that allows them to outcompete invading bacteria despite the large mutation load within their genomes. This raises the possibility that some of the non-synonymous mutations that rose to high frequency during the bottleneck may have allowed rapid adaptation, in other words a form of evolution by shifting balance^33^.

*H. pylori* seems to be an outlier amongst bacteria in many features of its biology^30^, including its slow rate of spread between human populations and its high mutation^28^ and recombination rates^29^. Our results suggest that recombination may save strains from rapid mutational meltdown but that deleterious mutations persist within populations, with the effects of bottlenecks enduring for millennia. The unusual properties of *H. pylori* make it a powerful model system for understanding how deleterious mutations interact with demographic processes and adaptive ones to mold diversity within natural populations.

## Methods

### Dataset collection

A dataset of 716 *Helicobacter pylori* whole-genome sequences was assembled, consisting of 213 newly sequenced isolates from Europe, Asia and Africa (Supplementary Data 1) and selected publicly available genomes (Supplementary Data 2). To complement the publicly available data, we included isolate collections from the following three main geographical areas: Europe, The Middle East, North East Africa and South East Asia. This new dataset is summarized in (Supplementary Data 1). Ethical permission for the collection of human gastric biopsy material had been obtained for all cohorts, including informed consent from the participating individuals. For details on the board/committee and institution that approved each study protocol, see Supplementary Data 3. The European and Middle Eastern genomes were included to obtain a more comprehensive mapping of ancestries within the area, the genomes from Central and North East Africa to have more thorough whole-genome representation of the “Ancestral Europe2” population as described previously^3^. Last, the South East Asian genomes were chosen due to their unadmixed hpAsia2 background to serve as donors for hpAsia2 ancestry. For details on sample collection and bacterial isolation in the different cohorts, see Supplementary Methods.

### Genome sequencing and annotation

New genomes were sequenced at five different centers: Karolinska Institute, Sweden (KI), Hannover Medical School, Hannover, Germany (MHH), Hellenic Pasteur Institute, Greece (HPI), Oita University, Japan (OiU), and University of Bath, UK (UBa) (Supplementary Data 1). For details on DNA extraction, library preparation, sequencing and primary bioinformatics, see Supplementary Methods.

Annotation of both newly sequenced draft genomes and publicly available sequences was performed using the prokka annotation pipeline v. 1.12^34^ using the most recent version of the 26695 annotation^35^ as primary annotation source.

Genome size and contig/scaffold number was collected from the prokka annotation output using the MultiQC tool^36^ and collected into Supplementary Data 4. All newly sequenced genomes were submitted to GenBank under BioProject PRJNA479414.

All strains, their population designations and their role in the respective analyses are shown in Supplementary Data 5.

### Sequence comparison and alignment

All isolates were mapped to the 26695 genome (NC000915.1) using the Snippy software v. 3.2-dev (https://github.com/tseemann/snippy). The resulting core genome, which was collected with the same tool, contained 287,746 core SNPs from 979,771 variant sites.

### FineSTRUCTURE

We inferred population structure among the strains based on the genome-wide haplotype data of the reference-based alignment to 26695 described above, using chromosome painting and fineSTRUCTURE^37^ v. 0.02 according to a procedure of our preceding study that applied them to *H. pylori* genome^38^. Briefly, we used ChromoPainter v. 0.04 to infer chunks of DNA donated from a donor to a recipient for each recipient haplotype and summarized the results into a “co-ancestry matrix”, which contains the number of recombination-derived chunks from each donor to each recipient individual. We then ran fineSTRUCTURE for 100,000 iterations of both the burn-in and Markov Chain Monte Carlo (MCMC) chain, in order to conduct clustering of individuals based on the co-ancestry matrix.

### Choice of donor and recipient strains and chromosome painting

D-statistics were calculated for strains assigned to each of the five ancestral populations hspEAsia, hpAsia2, hspCNEAfrica, hspENEAfrica and hspAfrica1WAfrica. D-statistics were calculated using popstats (https://github.com/pontussk/popstats) and specifying individual A as SouthAfrica7 (hpAfrica2), individual B as GAM260Bi (hspAfrica1WAfrica), individual Y as F227 (hspEAsia) and individual X. In this comparison, negative D-statistics imply more African ancestry in the strain designated as individual X than in F227. D-statistics values can be found in Supplementary Data 6 and 7.

A subset of strains from each of the five ancestral populations were chosen as donors to get groups of similar size and were selected based on the fineSTRUCTURE analysis to get good representativeness over the donor populations. Some hpAsia2 strains showed signs of elevated African admixture based on negative D-statistics values and these strains were not selected as donors.

We conducted chromosome painting of 646 recipient strains (belonging to hspNEurope, hspCEurope, hspSWEurope, hspMiddleEast, hspENEAfrica, hspEAsia, hspCNEAfrica, hspAfrica1WAfrica, hpAsia2, and hpAfrica2). For this purpose, we used ChromoPainterV2 software^37^. For each recipient population, we calculated site-by-site average copying probability from each of the five donor populations. Gene-by-gene averages were also calculated by averaging of the sites in each gene for the 790 genes in the alignment. Regressions between gene-by-gene averages in different hpEurope subpopulation were calculated using the aq.plot() (mvoutlier package) function in R.

### Shared drift estimation

We used the chromosome painting analysis to investigate shared genetic drift profiles. We calculated a separate drift profile for each pair of hpEurope subpopulations, including a within-population profile. For each profile, we calculated separate drift values for each of the five ancestry components (with the components shown in separate triangles in Fig. 1b). For example, to calculate the shared drift profile of hspNEurope and hspSWEurope, we took each combination of pairs of strains from the two populations and asked whether they used donors from the same population at each site in the genome. We also tabulated whether they used exactly the same donor strain. The drift value for that pair of populations for that ancestry component is the ratio of shared donor to shared strain, summed over all pairs and sites in the genome.

### dN/dS calculations

From the fineSTRUCTURE analysis, a sub-dataset was collected consisting of the European strains assigned to the hpEurope populations, together with a representative selection of strains from the ancestral populations hpAfrica2, hspAfrica1WAfrica, hspCNEAfrica, hspENEAfrica, hpAsia2, and hspEAsia. The *H. acinonychis* genome was added to this dataset to provide an outgroup. For a detailed list of strains that were included in these analyses, see columns in Supplementary Data 2 and 5. dN/dS was estimated pairwise between these strains from core genome alignments using the method of Yang and Nielsen^39^, as implemented in PAML v. 4.7^40^.

To calculate the numbers of excess mutations observed in the Asian populations, the mean number of non-synonymous mutations was calculated for African and Asian populations, and the difference was then multiplied by a correction factor of 1.37 to account for the loss of some coding sites in the core genome alignment (1.11 Mb) compared with the reference genome (1.52 Mb).

### Ancestry and mutation score analysis

Using *H. acinonychis* as a reference, non-synonymous and synonymous mutations were called against representative strains from the African and Asian populations. For this analysis the ancestral populations were combined to reduce the ancestry components to either African (hpAfrica) or Asian (hpAsia). For each site, the frequencies of these mutations within each ancestral population were calculated to give a score between 0 and 1. These scores were then combined by subtracting the hpAsia scores from the hpAfrica scores to give a score between −1 and 1, where −1 = fixed in hpAsia and absent in hpAfrica, 0 = equal frequencies in hpAsia and hpAfrica, 1 = absent in hpAsia and fixed in hpAfrica. Thus, these scores represent the mutational load for each site in the ancestral populations. We then combined these site-by-site scores with the site-by-site chromosome painting data for the European populations so that each site had an estimate of ancestry component from both Asia and Africa, and a mutational score representing the mutational load in the ancestral populations.

We then computed linear regressions of hpAsian ancestry against mutation score for each European population, and we did this for the positive (excess African) and negative (excess Asian) mutation scores separately. To confirm that the regression slopes were not driven by a small number of outlier genes we conducted a gene-by-gene jackknife by repeating the regressions but removing all the sites from a single gene each time. From this distribution of slope values we calculated the pseudovalues as pseudovalue = slope-((*n* − 1)*(j_slope-slope)). We then calculated the mean and confidence limits of these pseudovalues as mean(pseudovalues) ± quantile(*p* = 0.05/2, df = *n* − 1) * se(pseudovalues). All data manipulation and statistical analysis was performed in R 3.6.1^41^. Packages from the tidyverse collection were used extensively, and ggplot2 was used for plotting.

### PCA

The PCA was calculated using the software PLINK v. 1.90^42^ on biallelic SNPS from the core genome CDS of *H. pylori*. The filtered SNPs were first LD pruned using the same software to get independent SNPs.

### Rate of non-adaptive non-synonymous substitutions relative to neutral divergence

The rate of non-adaptive non-synonymous amino-acid substitutions relative to neutral divergence was calculated based on the method of Galtier^20^ and implemented in the software GRAPES^20^ v. 1.1.1. The software was run with folded SFS obtained from the core CDS as input and no divergence data. The synonymous and non-synonymous folded SFS were obtained using the PopGenome library^43^ in R^41^, and the number of synonymous and non-synonymous sites was estimated based on the reference core genome sequence.

### Admixture graphs

Admixture graphs between the different populations were obtained using the software Treemix v. 1.12^12^. Treemix was run with a number of migration edges between 0 to 15, with 10 replicates for each number of edge and hpAfrica2 was set as the outgroup. The final number of migration edges was chosen as being the smallest number that allowed 99.8% of the variance to be explained, which is the same criterion used by ref. 12.

### Simulation of bacterial populations evolving under deleterious mutation pressure

We used SLiM v. 3.6^44^ to simulate the accumulation of deleterious mutations in bacterial populations. Each bacterial genome was a single haploid chromosome of length 1.6 Mb, with a mutation rate per site per generation of 5 × 10^−7^. This corresponds to about 1/20 of the mutation rate per year estimated from *H. pylori* data^28,45^. Half of the mutations were neutral, with the other half being deleterious with six different selection coefficients (−5 × 10^−3^, −2 × 10^−3^, −10^−3^, −5 × 10^−4^, −2 × 10^−4^, −10^−4^), each accounting for 1/12 of the mutations. There was no back mutation and mutations had a multiplicative effect on fitness.

At the beginning of the simulation, there was no variation in the population. These parameters resulted in an intermediate mutation load for each strain. Higher mutation rates would have led to excessive load in each bacterial generation, while smaller selection coefficients would have led to individual deleterious mutations behaving as if they were neutral.

In each generation, recombination happened between strains from the same population via the transfer of one segment from a donor strain into a recipient strain, with the length of the block taken from an exponential distribution. We used three different mean values, 0, 5000 and 50,000 bp. *H. pylori* has a high rate of import of tracts with mean around 400 bp^46^ but simulating larger tracts is more computationally efficient than simulating many tracts.

A single population of size 10,000 evolved for 5000 generations, by which time the population was in approximate mutation-selection equilibrium. In total, 1000 strains were moved into a second population, which retained this size for 500 generations before expanding to size 10,000. This is substantially below estimated ancestral population sizes of 2,000,000 or more for *H. pylori*^6^ but sizes substantially in excess of this are difficult to simulate due to memory and computation time issues. From generation 8000 onwards, migration from the non-bottleneck to the bottleneck populations happened at a rate of one strain every 500 generations. The simulations were completed after 15,000 total generations.

Simulation output was analyzed using python and R. For each population, we calculated its average fitness, its fitness variance, its dN/dS to the ancestor and within population dN/dS. Subsequent to admixture, the ancestry proportion from each population was tabulated based on mutations that had arisen during the separate evolution of the populations prior to mixture. For sites without such mutations, their origin was assumed to be the same as the closest mutation of known ancestry, provided that the mutation was within 1 kb. Otherwise, its origin was recorded as unknown.

The SLiM, python and R scripts can be found at https://github.com/EliseTourrette/Hpylori/tree/main/HpEurope.

### Reporting summary

Further information on research design is available in the Nature Portfolio Reporting Summary linked to this article.

## Supplementary information

Supplementary information

Description of Additional Supplementary Files

Supplementary Data 1–8

Reporting Summary

## Data Availability

All newly sequenced genomes can be found in GenBank (https://www.ncbi.nlm.nih.gov/genbank/) under BioProject PRJNA479414. Individual accessions of the genomes can be found in Supplementary Data 1. For the publicly available genomes, GenBank accessions or equivalents in other databases can be found in Supplementary Data 2. The entire dataset of 716 genomes has been deposited in Data Dryad, accessible here: 10.5061/dryad.v9s4mw70c. Source data are provided with this paper.

## Source data

Source Data

## Code availability

The SLIM simulation scripts have been deposited on GitHub^47^: https://github.com/EliseTourrette/Hpylori/tree/main/HpEurope (10.5281/zenodo.7130003).
