# Author Correction: Repeated out-of-Africa expansions of *Helicobacter pylori* driven by replacement of deleterious mutations

**DOI:** 10.1038/s41467-023-37302-5

**Published:** 2023-03-20

**Authors:** Harry A. Thorpe, Elise Tourrette, Koji Yahara, Filipa F. Vale, Siqi Liu, Mónica Oleastro, Teresa Alarcon, Tsachi-Tsadok Perets, Saeid Latifi-Navid, Yoshio Yamaoka, Beatriz Martinez-Gonzalez, Ioannis Karayiannis, Timokratis Karamitros, Dionyssios N. Sgouras, Wael Elamin, Ben Pascoe, Samuel K. Sheppard, Jukka Ronkainen, Pertti Aro, Lars Engstrand, Lars Agreus, Sebastian Suerbaum, Kaisa Thorell, Daniel Falush

**Affiliations:** 1 Department of Biostatistics, University of Oslo, Oslo, Norway; 2 CAS Key Laboratory of Molecular Virology and Immunology, Institut Pasteur of Shanghai, Chinese Academy of Sciences, Shanghai, China; 3 Antimicrobial Resistance Research Center, National Institute of Infectious Diseases, Tokyo, Japan; 4 Pathogen Genome Bioinformatics and Computational Biology, Research Institute for Medicines (iMed.ULisboa), Faculty of Pharmacy, Universidade de Lisboa, Lisbon, Portugal; 5 University of Chinese Academy of Sciences, Beijing, China; 6 National Reference Laboratory for Gastrointestinal Infections, Department of Infectious Diseases, National Institute of Health Dr Ricardo Jorge, Lisbon, Portugal; 7 Department of Microbiology, Hospital Universitario La Princesa, Instituto de Investigación Sanitaria Princesa, Madrid, Spain; 8 Gastroenterology Laboratory, Rabin Medical Center, Petah Tikva, Israel; 9 Department of Digital Medical Technologies, Holon Institute of Technology, Holon, Israel; 10 Department of Biology, Faculty of Sciences, University of Mohaghegh Ardabili, Ardabil, Iran; 11 Department of Environmental and Preventive Medicine, Oita University Faculty of Medicine, Yufu, Oita Japan; 12 Department of Medicine-Gastroenterology, Baylor College of Medicine, Houston, TX USA; 13 Laboratory of Medical Microbiology, Hellenic Pasteur Institute, Athens, Greece; 14 G42 Healthcare, Abu Dhabi, UAE; 15 Elrazi University, Khartoum, Sudan; 16 Department of Biology, University of Oxford, Oxford, UK; 17 Ineos Oxford Institute, Department of Biology, University of Oxford, Oxford, UK; 18 Center for Life Course Health Research, University of Oulu, Oulu, Finland; 19 Primary Health Care Center, Tornio, Finland; 20 Arokero Oy, Tornio, Finland; 21 Center for Translational Microbiome Research, Department for Microbiology, Tumor, and Cell Biology, Karolinska Institutet, Stockholm, Sweden; 22 Division of Family Medicine, Karolinska Institutet, Stockholm, Sweden; 23 Department of Medical Microbiology and Hospital Epidemiology, Max von Pettenkofer Institute, Faculty of Medicine, LMU Munich, Munich, Germany; 24 Department of Medical Microbiology and Hospital Epidemiology, Hannover Medical School, Hanover, Germany; 25 DZIF German Center for Infection Research, Hannover-Braunschweig and Munich Partner Sites, Munich, Germany; 26 Institute of Biomedicine, Department of Infectious Diseases, University of Gothenburg, Gothenburg, Sweden; 27 Department of Clinical Microbiology, Sahlgrenska University Hospital, Gothenburg, Sweden

**Keywords:** Genetic variation, Bacterial genomics

Correction to: *Nature Communications* 10.1038/s41467-022-34475-3, published online 11 November 2022

In this article, the acknowledgement to the National Institute of Genetics and the Human Genome Center at the Institute of Medical Science (University of Tokyo) was accidentally omitted.

The original article has been corrected.

