# Supplementary material for: Repeated out-of-Africa expansions of Helicobacter pylori driven by replacement of deleterious mutations
Source: Nat Commun. 2022 Nov 11;13:6842. doi: 10.1038/s41467-022-34475-3 (PMC9652371; doi:10.1038/s41467-022-34475-3)
Supplement: Supplementary file 1 — Supplementary information [file 41467_2022_34475_MOESM1_ESM.pdf]

Repeated out-of-Africa expansions of *Helicobacter pylori* driven by replacement of deleterious mutations

Supplementary figures

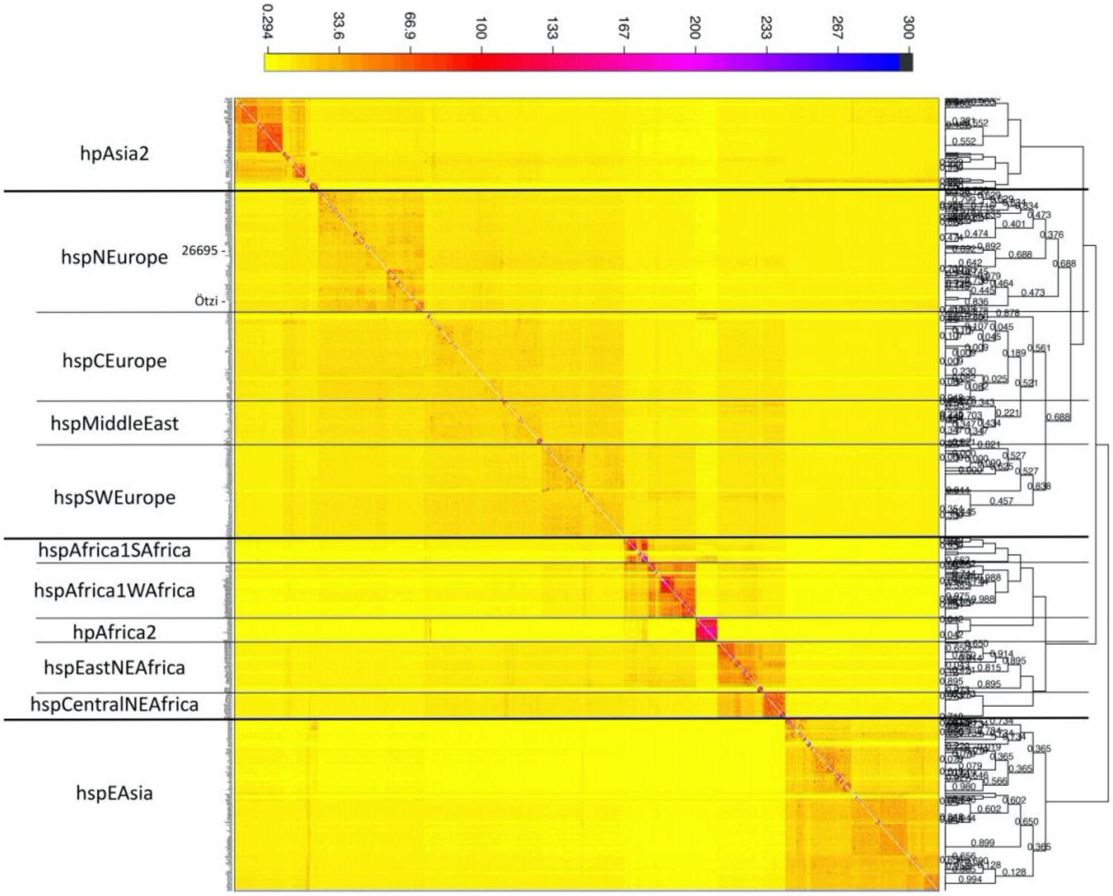

Figure S1: heatmap showing populations inferred by fineSTRUCTURE. For each individual, the number of chunks donated by other individuals are shown according to the colour scale at the top. Note the position of Ötzi within hpsNEurope. The tree on the right shows hierarchical clustering of fineSTRUCTURE populations.

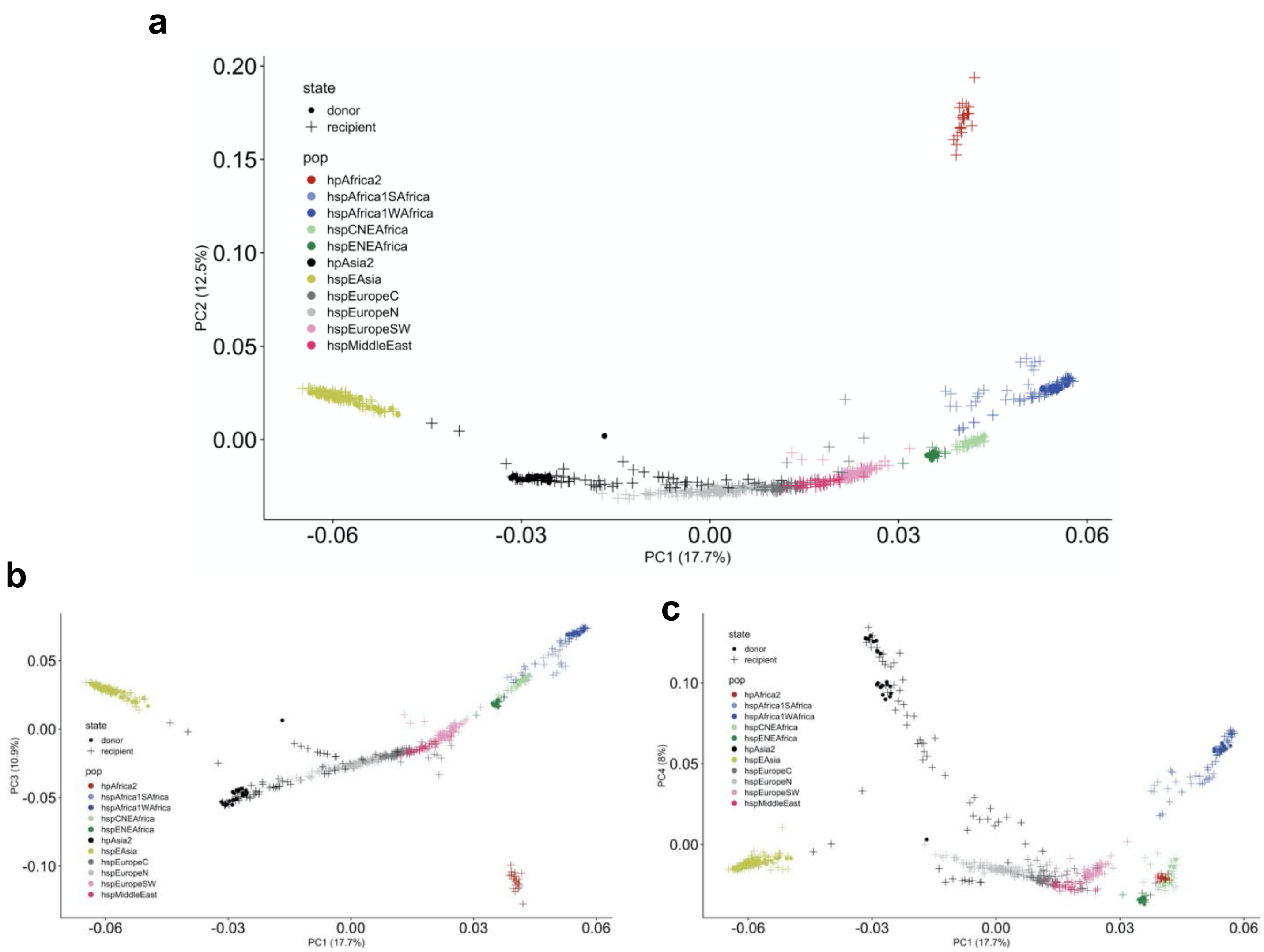

Figure S2: Principal Component Analysis (PCA) of the *Helicobacter pylori* strains used. The first four components are shown, and the strains are colored based on their populations. Strains used as donors in the fineSTRUCTURE analysis are shown as circles while the recipient are shown as crosses. (a) PC2 vs PC1, (b) PC3 vs PC1 and (c) PC4 vs PC1.

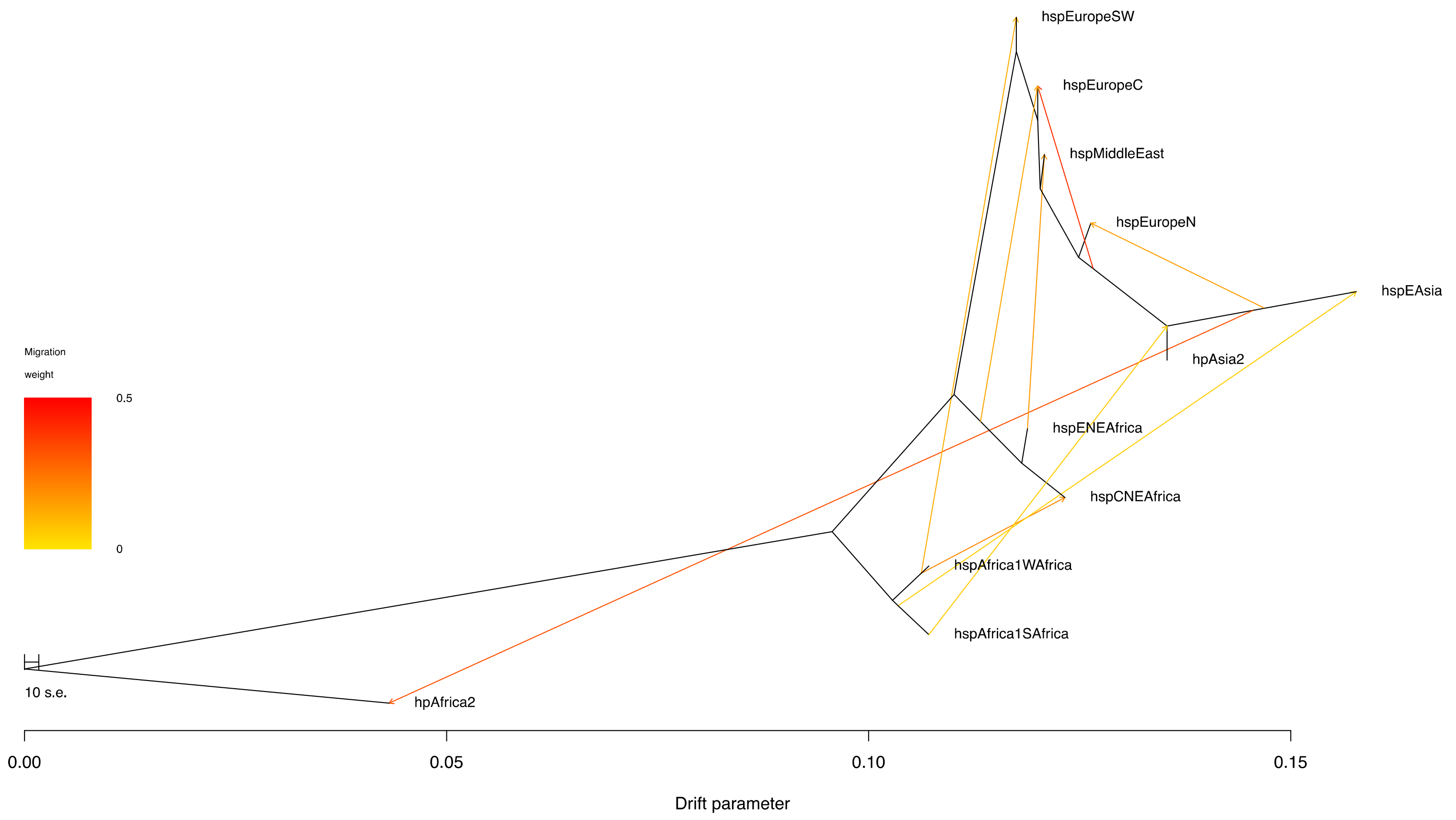

Figure S3: Admixture graph for the different populations. Nine migration edges – the optimum number of migration edges are shown. The population hpAfrica2 was set as the outgroup. The arrows represent the gene flows between the different branches, their color indicating the weight of the migration edge.

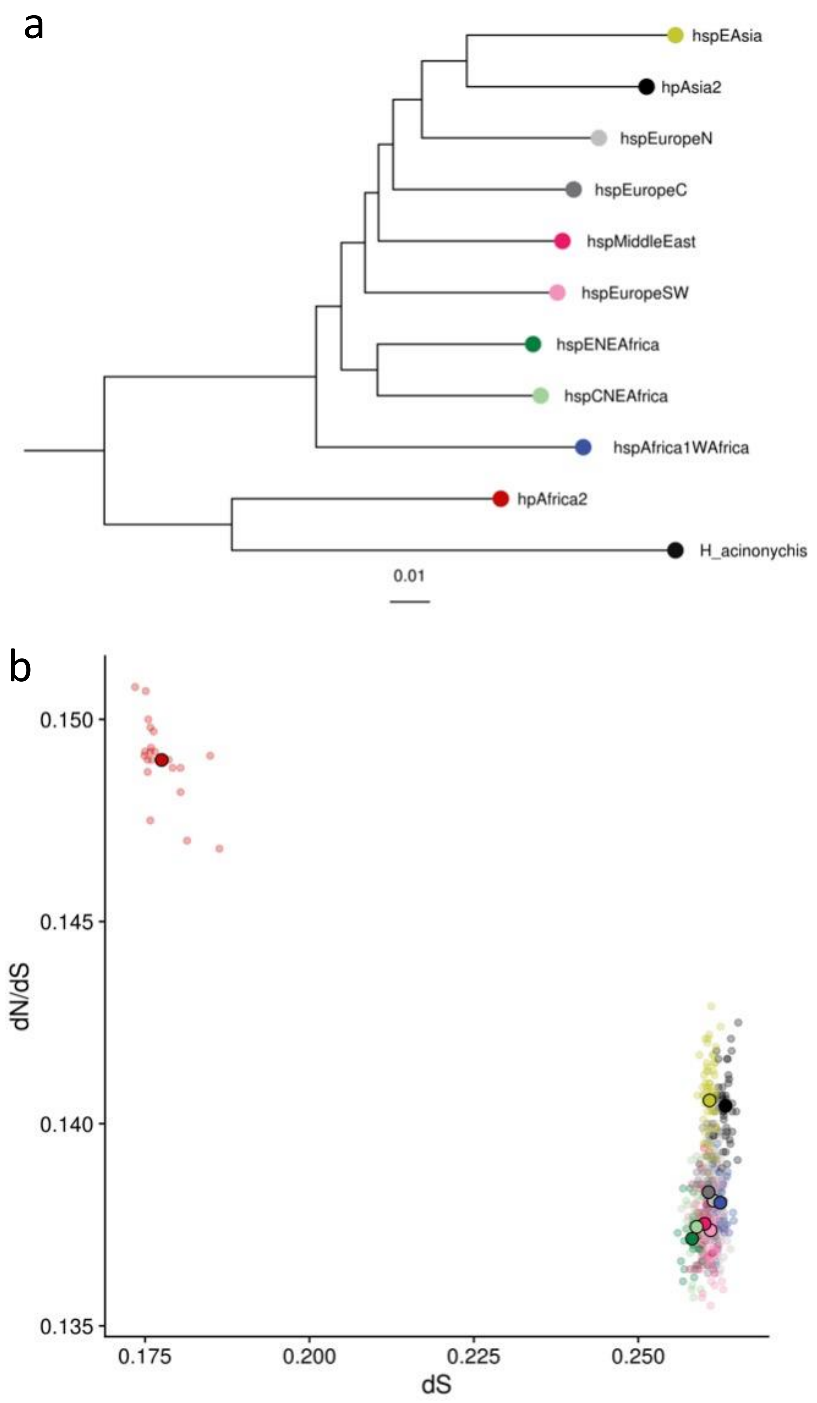

Figure S4: Relationships amongst populations. (a) Neighbor joining tree based on genetic distances between populations. (b) dN/dS plotted against dS to *H. acinonychis* outroups for all populations, including Africa2.

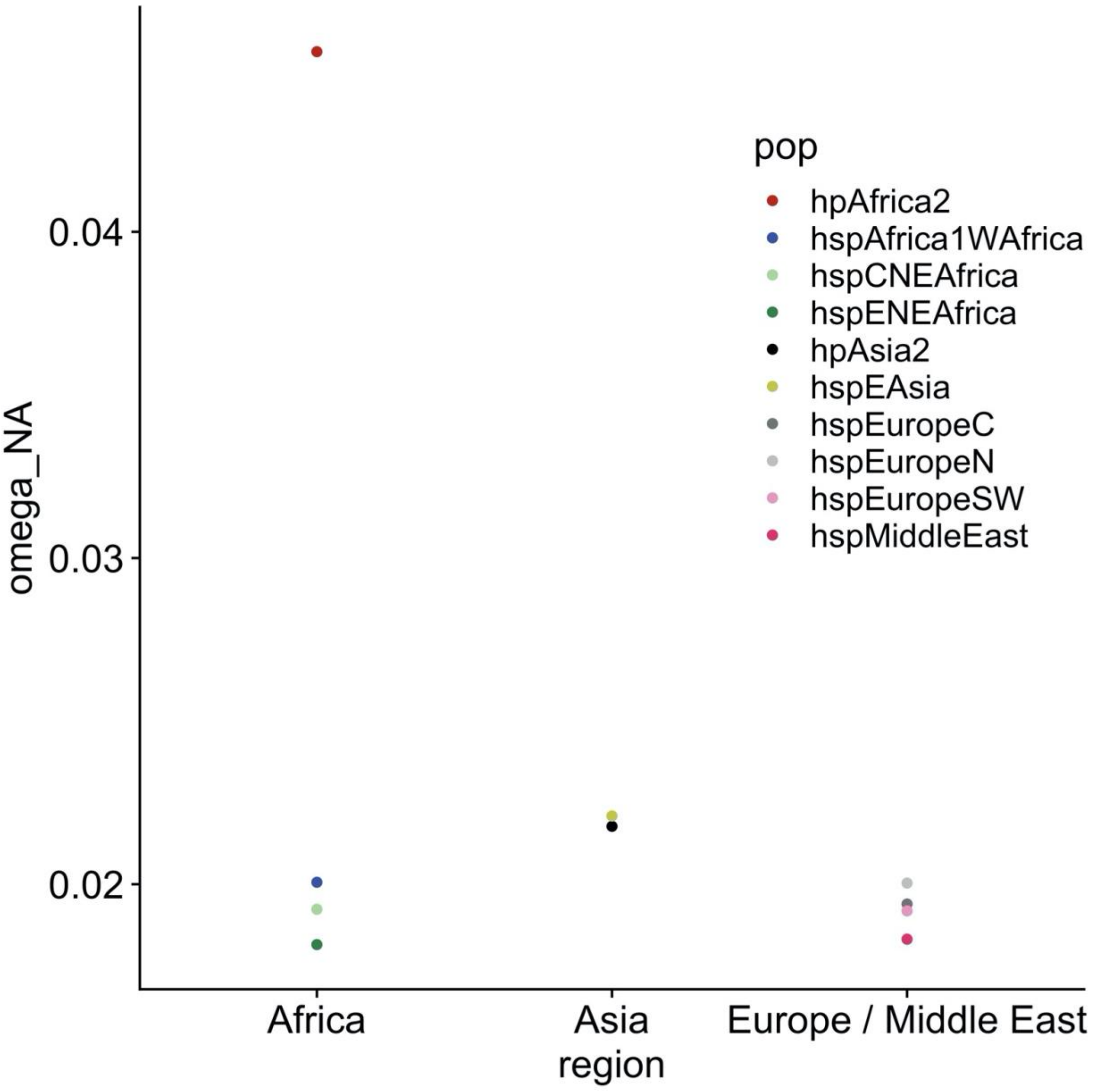

Figure S5: Rate of non-adaptive non-synonymous amino-acid substitutions relative to neutral divergence. The different subpopulations are separated based on the different geographical regions on the x-axis.

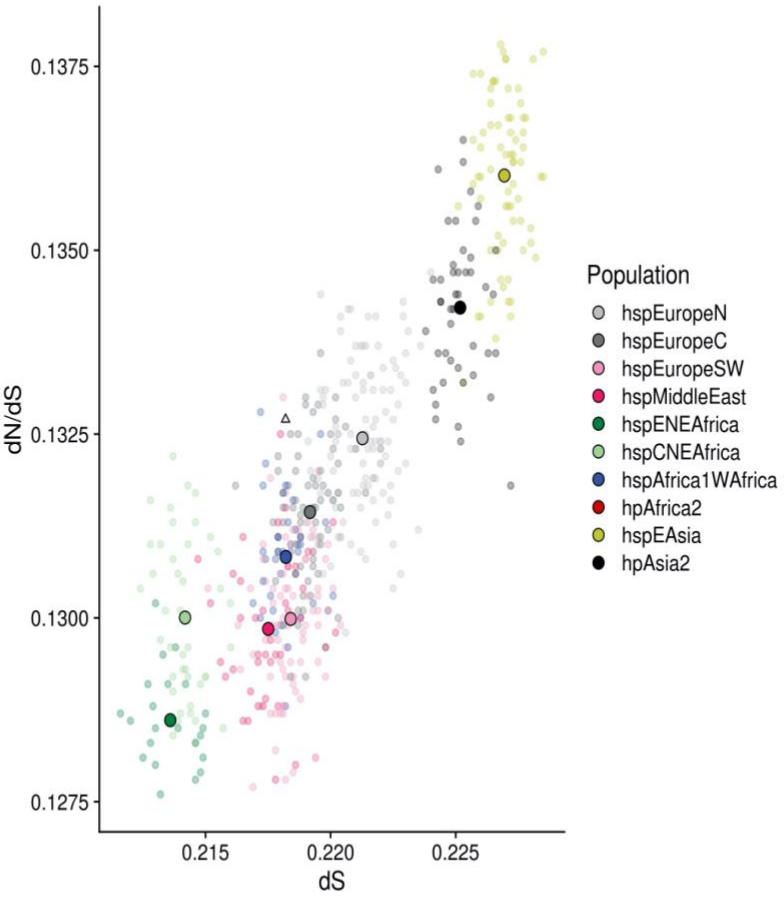

Figure S6: Between population divergence.
dN/dS calculated using hpAfrica2 as an outgroup, plotted against dS for isolates (semi opaque points) and populations (solid points), excluding hpAfrica2 isolates.

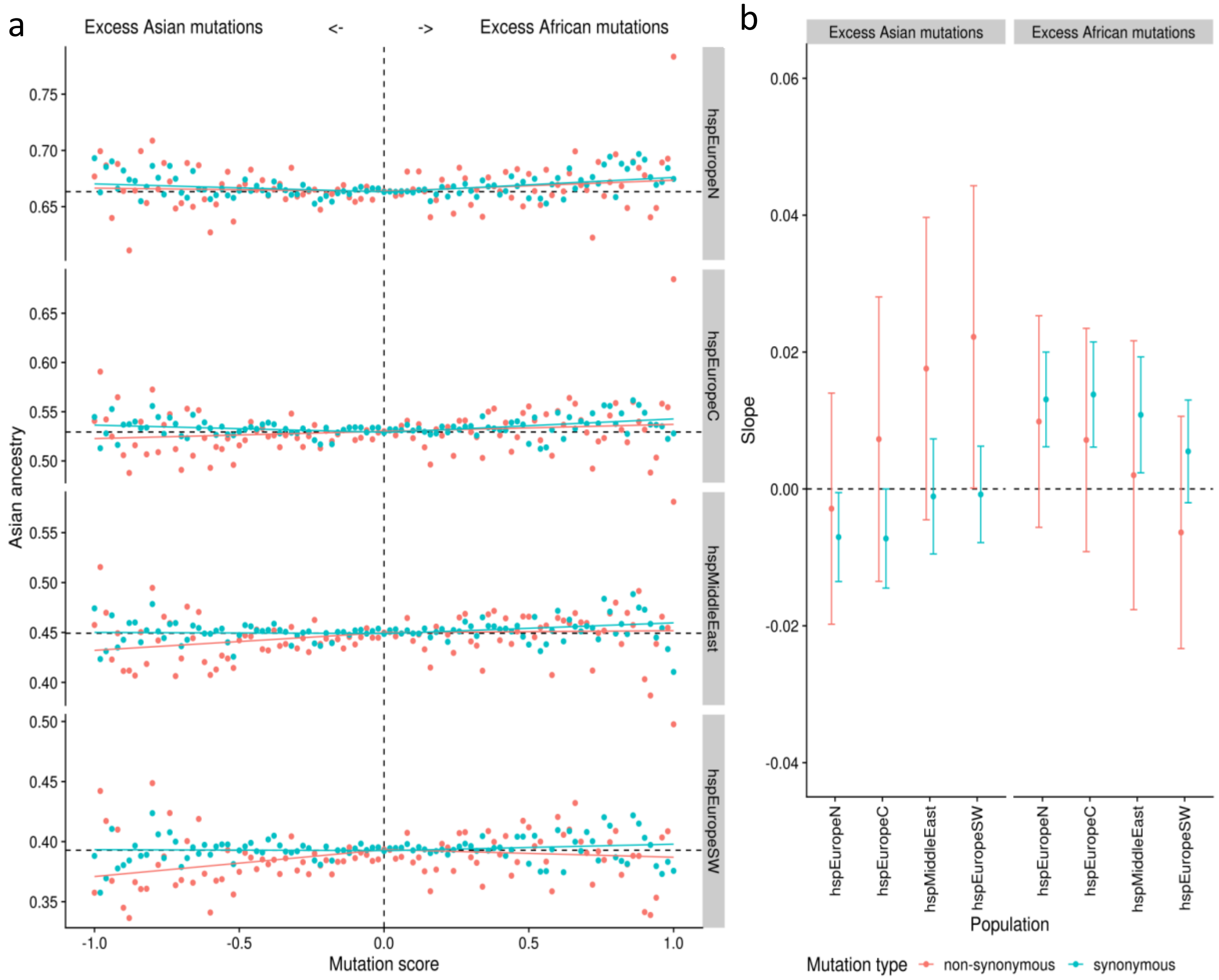

Figure S7: Genetic ancestry of hpEurope subpopulations as a function of mutation score. (a) Average ancestry in chromosome painting analyses plotted against mutation score (mutation frequency in African populations minus mutation frequency in Asian populations) in bins of 0.02. Regression lines were calculated separately for positive and negative mutation scores. (b) Regression slopes with 95% confidence intervals estimated using a gene-by-gene jackknife for excess Asian and excess African mutations, respectively.
hpAfrica2 was used as an outgroup.

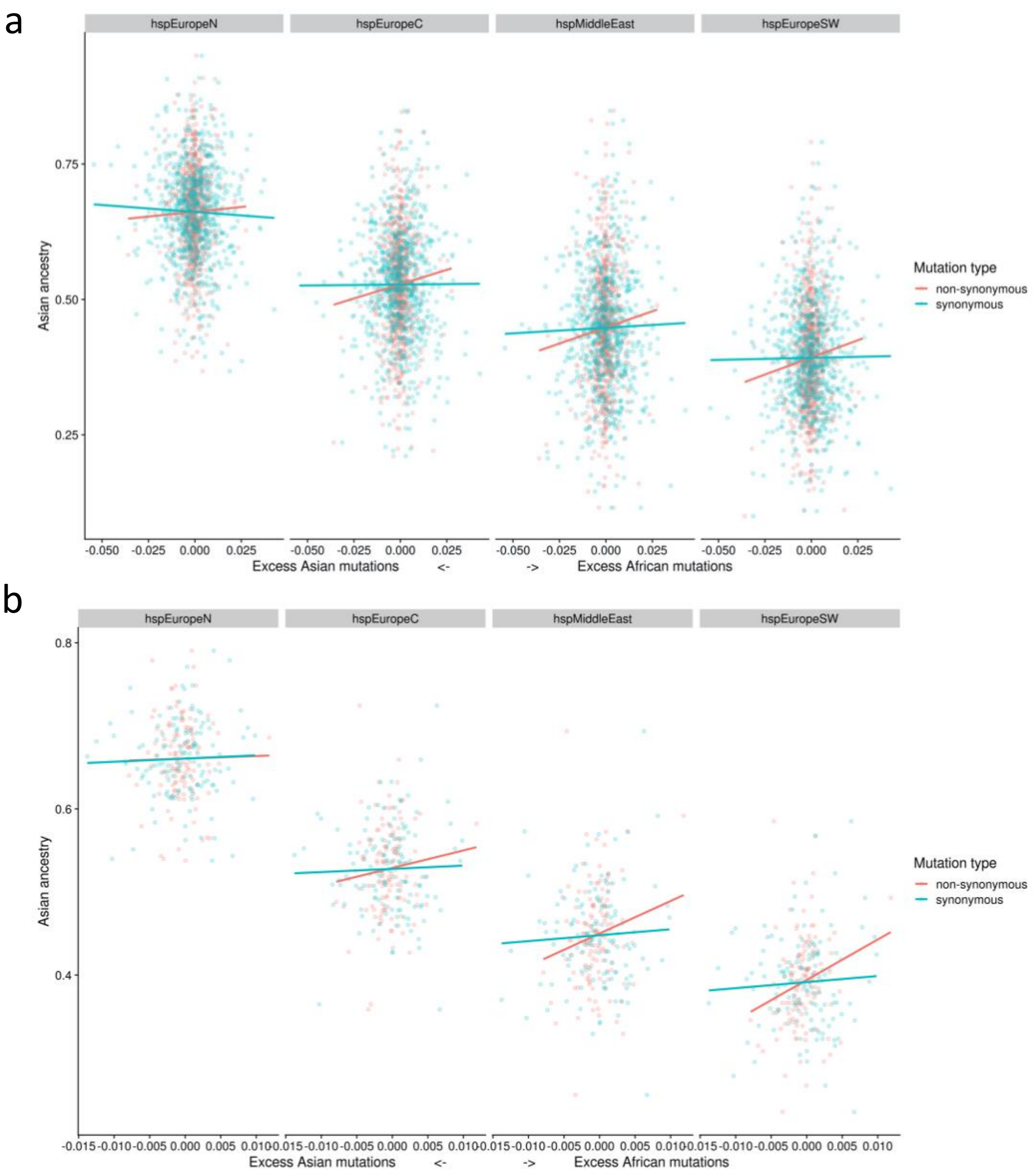

Figure S8: Average ancestry in chromosome painting analyses plotted against mutation score (mutation frequency in African population minus mutation frequency in Asian population) (a) by genes and (b) by 10 kb bins. In both cases, only the regression in hspEuropeSW, for the non-synonymous sites, is significant.

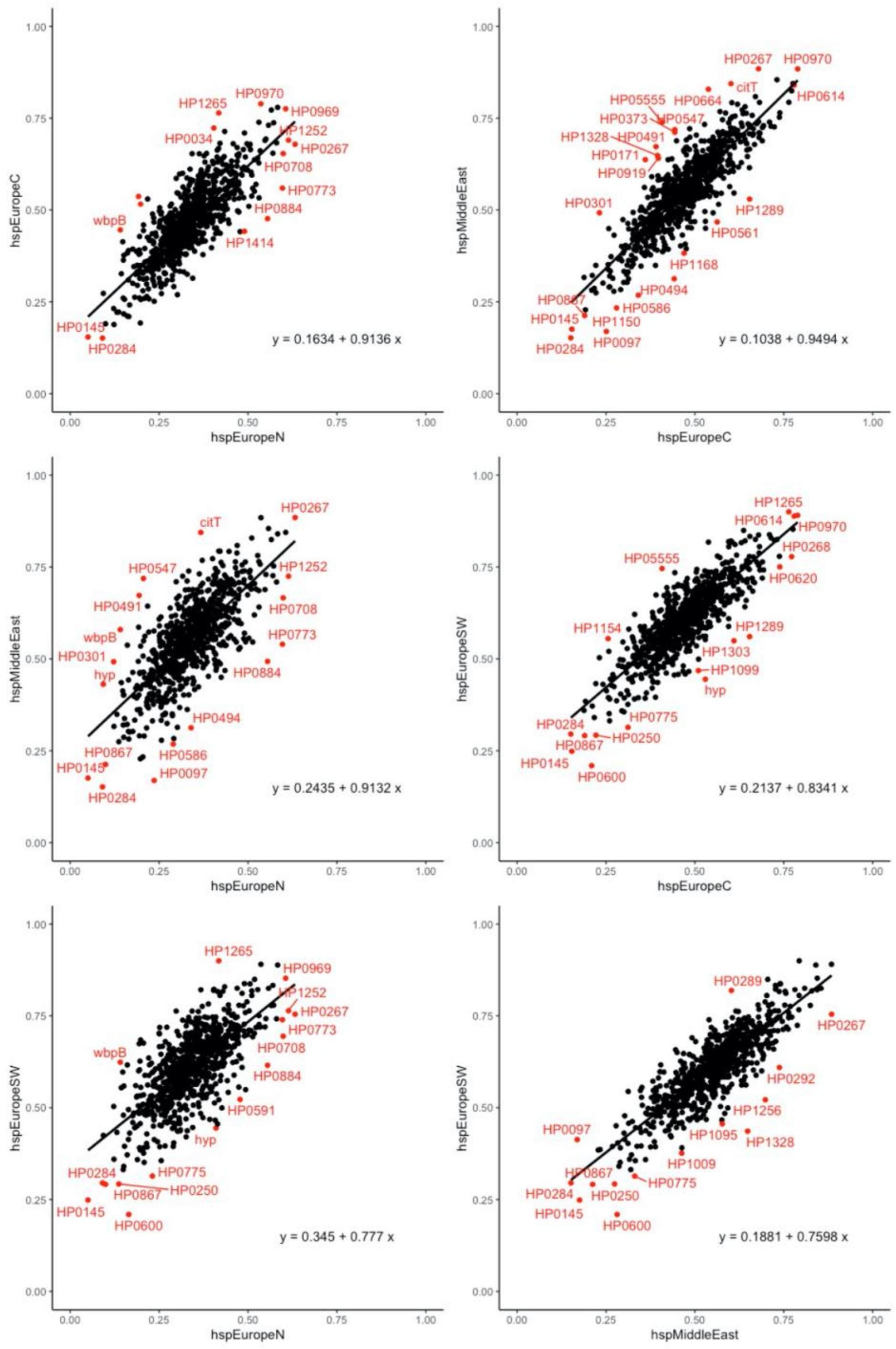

Figure S9: Average African ancestry proportions of genes.

Each panel shows the relationship between the proportion of ancestry assigned to Africa in the chromosome painting for each gene, for pairs of hpEurope subpopulations. Outliers from the regression slope are calculated based on robust Mahalanobis distances using the R function aq.plot(data, quan =1,alpha =0.015).

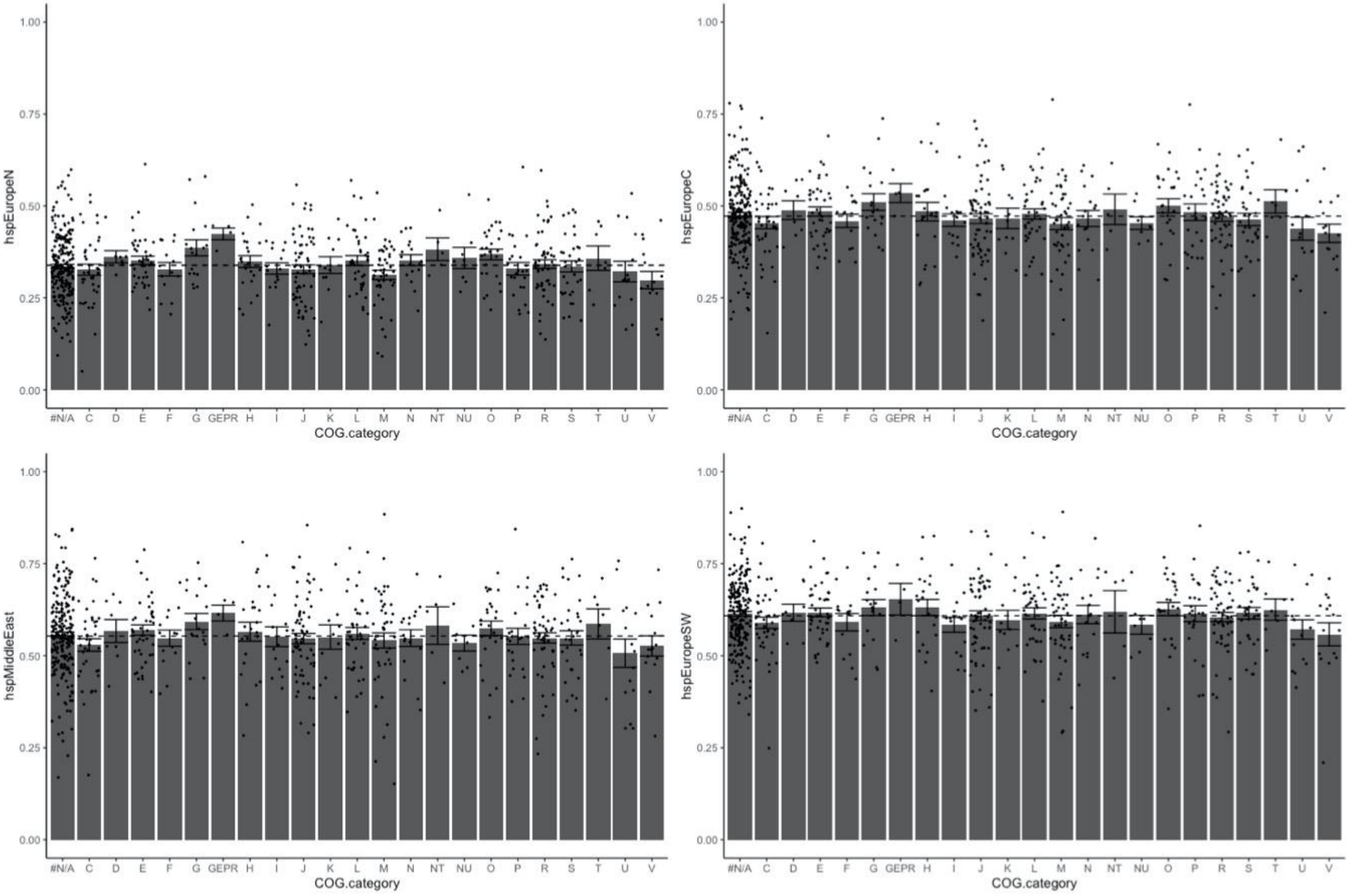

Figure S10: Average African ancestry proportion of genes in particular COG category.
Calculated separately for each hpEurope subpopulation. Whiskers show standard error of the average for each category (the number of genes for each category is $n_{\#N/A} = 226$, $n_C = 38$, $n_D = 8$, $n_E = 41$, $n_F = 14$, $n_G = 18$, $n_{GEPR} = 4$, $n_H = 23$, $n_I = 15$, $n_J = 65$, $n_K = 11$, $n_L = 39$, $n_M = 44$, $n_N = 17$, $n_{NT} = 5$, $n_{NU} = 8$, $n_O = 26$, $n_P = 24$, $n_R = 51$, $n_S = 32$, $n_T = 7$, $n_U = 14$, $n_V = 14$).

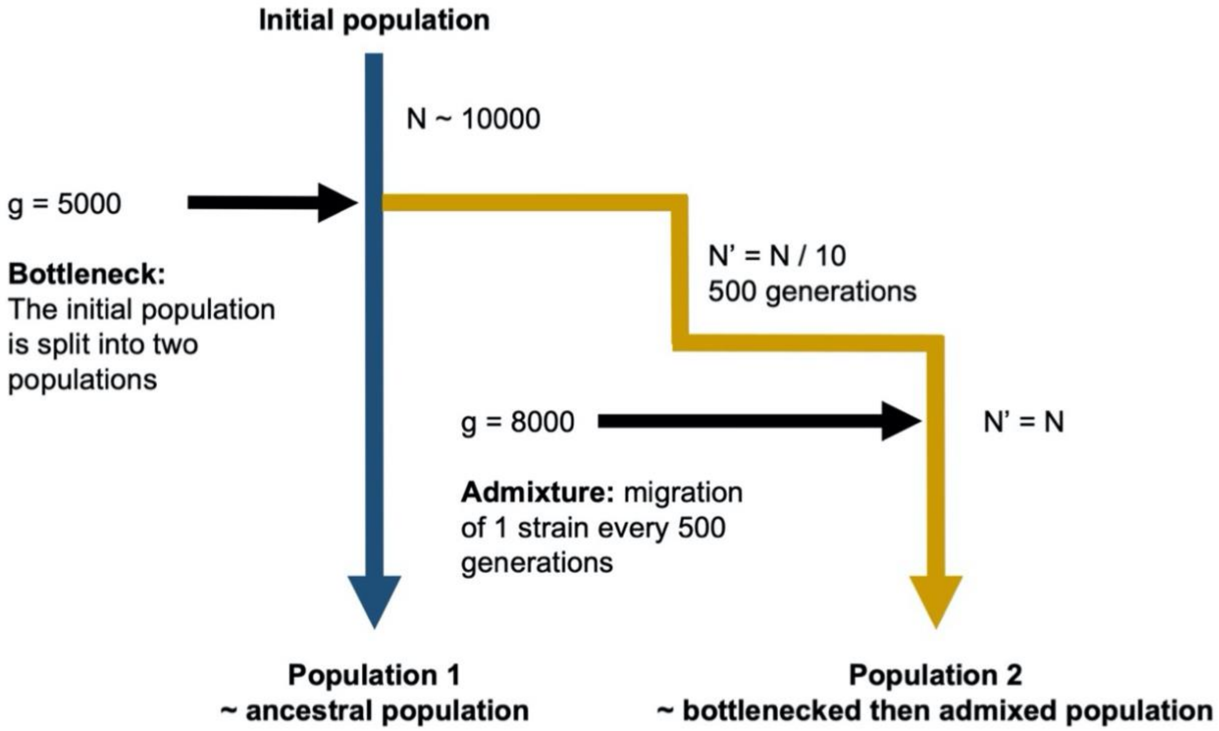

Figure S11: The different steps of the simulation process.
Parameters values: N = 10000; genome length = 1.6 Mb; mutation rate = $5x10^{-7}$ per bp per generation; deleterious mutations: s = (-0.005, -0.002, -0.001, -0.0005, -0.0002, -0.0001) and they represent 50% of the mutations (the other 50% are neutral mutations). Look at different recombination levels: clonal reproduction (import size per generation = 0bp), intermediate recombination levels (import size per generation = 500bp and 5000bp on average) and nearly free recombination (import size per generation = 50000bp on average).

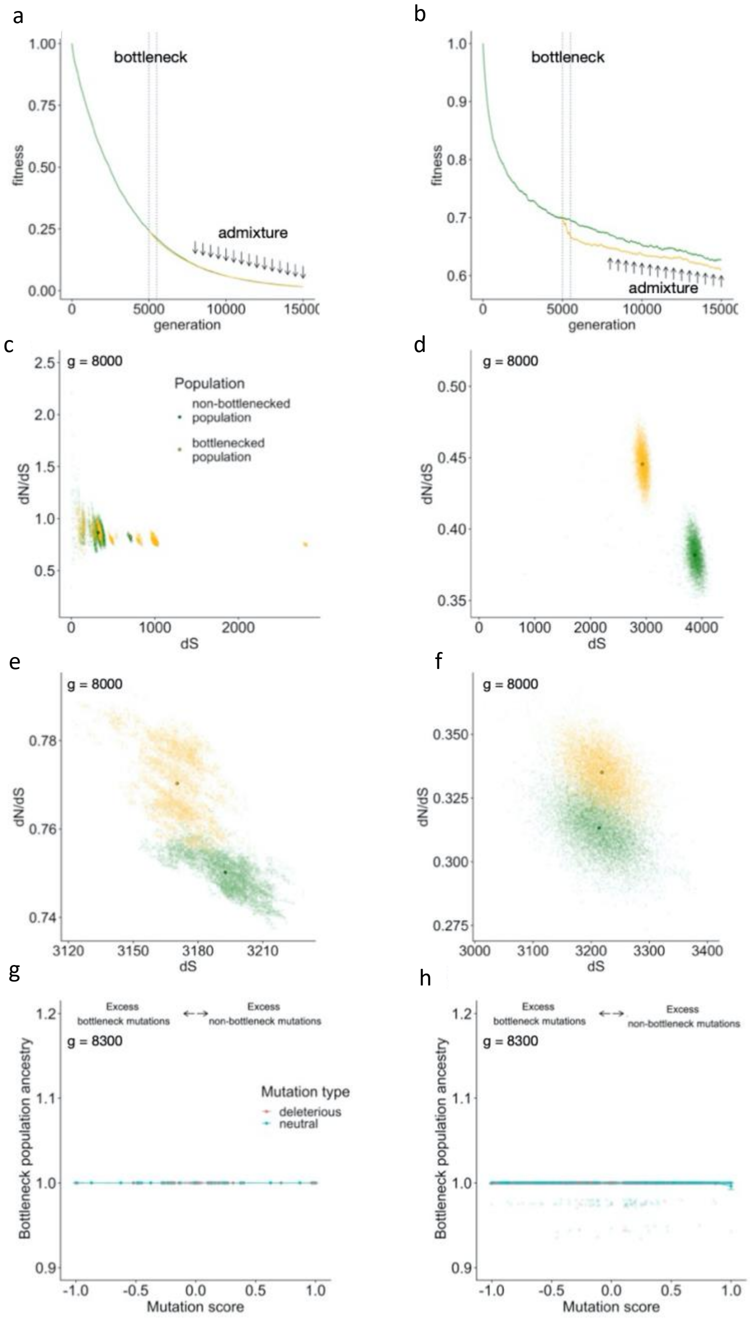

Figure S12: The effect of different recombination rates.
(a-b) Average fitness over the generations. (c-d) Within population dN/dS (y axis) plotted against dS (x axis). Semi opaque points show pairwise distances; solid points indicate population means. (e-f) dN/dS calculated to the ancestor plotted against dS for isolates (semi opaque points) and populations (solid points). (g-h) Average bottleneck population ancestry, after the first migration event, plotted against mutation score (frequency in the non-bottleneck population minus the frequency in the bottleneck population before the admixture begins), under no recombination (a,c,e,g) and high recombination (b,d,f,h). The segment represents the bottleneck and the arrows signal the migration events.

Supplementary Methods

Isolate collection

For a summary of the origin, cohorts and ethical permissions of the isolates sequenced within this project, see Supplementary Data 3.

Swedish (Kx-*) isolates were collected within the Kalixanda study and were randomly selected from this northern Swedish cohort. The cohort and sampling procedure[1] as well as the *H. pylori* isolation and culture[2] has been described previously. Briefly, biopsies were placed in freezing medium with 10% glycerol and frozen immediately at -20°C after endoscopy and moved to -70°C within 2 weeks. Primary cultures were plated on blood agar with Skirrow supplement and subsequent passages on Columbia agar with 8.5% horse blood and 10% horse serum. All cultures were performed at 37°C in 5% oxygen. *H. pylori* colonies were identified by microscopy and urease, catalase and oxidase tests. Both the homogenized biopsy in the freezing medium as well as the propagated single colonies were stored in -80°C until further use.

UK isolates were collected as part of a previous sampling effort, with sampling methods and ethical approval previously described in Berthenet et al., 2018 [3]. Briefly, samples were obtained from patients attending for upper GI endoscopy at Nottingham University Hospitals NHS Trust or Swansea Bay University Health Board Hospitals. Bacteria were sampled from patients by gastric biopsy and grown on *H. pylori*-selective medium (Dent plates) at 37°C in a microaerophilic environment (CampyGen or microaerophilic cabinet) for 5 to 10 days. Informed consent was obtained from participants by the appropriate health board.

Belgian isolates were collected as described previously in Berthenet et al. 2018 [3].

Portuguese isolates: Clinical strains were isolated by culture from an antral biopsy specimen, at the national reference laboratory from the National Institute of Health Dr Ricardo Jorge. Briefly, biopsy specimens were sampled for diagnostic purposes and immediately placed into a transport medium. At the laboratory, the biopsies were macerated and plated into an *H. pylori*-specific medium (*Pylori* gelose, bioMérieux, Marcy l'E´toile, France). The plates were incubated at 37°C for 4-7 days under microaerobic conditions using the anaerobic cultivation system Anoxomat (Anoxomat, Mart). All strains were kept at -80°C, in glycerol 20%. For the present study, *H. pylori* strains from different years (1990, 1992, 1995, 1999-2000, 2002, 2004-2005, 2007 and 2009-2013) were included. Ethical approval for anonymized use, for research purposes, of clinical strains from the collection of the reference laboratory was obtained from the Health Ethics Commission from the National Institute of Health Dr Ricardo Jorge. The Portuguese strains prefixed "Pt" were previously published by Vale et al. 2017 [4].

German and French isolates, as well as the Swedish isolates prefixed "Sw-" were also previously described by Vale et al. 2017 [4].

Greek isolates: Antral mucosa biopsy specimens collected from the greater curvature were aseptically placed in thioglycolate medium (Oxoid, Basingstoke, United Kingdom) and were processed for *H. pylori* isolation within 2 to 4 h after endoscopy. Specimens were vigorously vortexed with addition of sterile glass beads and cultured for up to 7 days on Chalgren-Wilkins agar plates containing antibiotics (vancomycin, 10 g/ml; trimethoprim, 10 g/ml; polymyxin B, 104 IU/liter; amphotericin B, 2 g/ml; nalidixic acid, 10 g/ml; bacitracin, 30 g/ml; and fluorocytosine, 5 g/ml) supplemented with 7% (vol/vol) horse blood and 1% (vol/vol) Vitox (Oxoid, Basingstoke, United Kingdom) under microaerophilic conditions (CampyPak Plus; Becton Dickinson, Cockeysville, MD) at 37°C. Plates were inspected daily for the presence of suspected colonies, which were initially screened for by colony morphology analysis and Gram staining and further verified by oxidase, catalase, and urease reactions. Culture sweeps, as well as individual colonies from each patient, were collected. *H. pylori* clinical isolates collected, were stored in brain heart infusion broth supplemented with 20% glycerol at 80°C until further analysis.

Israeli isolates: All biopsies were obtained from patients underwent gastroscopy and routinely tested for *H. pylori* antibiotic resistance due to gastritis and previous treatment failures as described previously by Boltin et al. [5]. Biopsy specimens were inoculated directly onto Columbia blood agar (Difco, Detroit, MI) supplemented with yeast extract (5 g/L), laked lysed horse blood (7%), vancomycin (3 mg/L), colistin sulfate (7.5 mg/L), nystatin (12500 IU/L), and co-trimoxazole (5 mg/L). Cultures were incubated for 72 h at 37°C under microaerophilic conditions. *H. pylori* isolates were identified by colony morphology, characteristic spiral morphology on Gram staining, and positive findings on catalase, urease, and oxidase test.

Iranian isolates were collected as previously described by Latifi-Navid et al., 2010 [6].

Cameroonian isolates were collected as previously described by Nell et al., 2013 [7].

Sudanese isolates: Gastric biopsy specimens were obtained from 200 consecutive patients undergoing diagnostic upper gastrointestinal endoscopy in four district hospitals in the Khartoum area. One endoscopic biopsy sample was obtained from the antral mucosa within a 5 cm radius of the pylorus. The samples were transported in aliquots containing 1.5 ml of brain heart infusion medium (Oxoid CM 225), yeast extract (Oxoid L21) and 20% glycerin in a cold box to the laboratory.

Nigerian isolates were previously reported by Linz et al., 2007 [8].

Bangladeshi isolates: The study enrolled patients who underwent endoscopy at Dhaka Medical College in November 2014 and has previously been described by Aftab et al. 2017 [9]. The collection and isolation procedure followed the same protocol as described for the Thai isolates below.

Thai isolates: The study enrolled patients who underwent endoscopy in Maesot city in November 2013 for Thailand. Exclusion criteria were the following: a history of partial gastric resection; previous eradication therapy for *H. pylori*; or treatment with bismuth-containing compounds, H2-receptor blockers, or proton pump inhibitors (PPI) in the previous four weeks. Experienced endoscopists collected gastric biopsy specimens during each endoscopy session, including two samples (for culture and histology) from the lesser curvature of the antrum approximately 3 cm from the pyloric ring and one sample from the greater curvature of the corpus (for histology).

For *H. pylori* culture, antral biopsy specimens were homogenized and inoculated onto antibiotics selection plates and were subsequently subculture on Mueller Hinton II Agar medium (Becton Dickinson, Franklin Lakes, NJ, USA) supplemented with 10% horse blood without antibiotics (Nippon Biotest Laboratories Inc., Tokyo, Japan). The plates were incubated for up to 10 days at 37°C under microaerophilic conditions (10% O_2, 5% CO_2, and 85% N_2). *H. pylori* isolates were identified based on colony morphology; Gram staining results; and oxidase, catalase, and urease reactions. Isolated strains were stored at −80°C in Brucella Broth (Difco, Franklin Lakes, NJ, USA) containing 10% dimethyl sulfoxide and 10% horse serum (Nippon Biotest Laboratories Inc., Tokyo, Japan).

Genome Sequencing

New genomes were sequenced at five different centres: Karolinska Institute, Sweden (KI), Hannover Medical School, Hannover, Germany (MHH), Hellenic Pasteur Institute, Greece (HPI), Oita University, Japan (OiU), and University of Bath, UK (UBa) (Supplementary Data 1).

Genomic DNA from strains marked with KI in Supplementary Data 1 was extracted using DNeasy Mini Kit (Qiagen, Hilden, Germany) following the manufacturer's guidelines for Gram-negative bacteria. Sequencing libraries were prepared using the TruSeq Nano kit (Illumina, San Diego, CA, USA) and sequenced on the MiSeq platform, v3 chemistry, using 300 bp paired end mode.

Genomic DNA from strains marked MHH was isolated from *H. pylori* strains after 24h culture on *H. pylori* selective agar (in-house recipe) with the Genomic Tip 100/G (Qiagen, Hilden, Germany). Nextera XT libraries were generated and sequenced in three different runs on MiSeq 2x300bp paired (Illumina, San Diego, CA, USA), as recommended by the manufacturer. All quantification steps of gDNA and NGS libraries were done with Qubit dsDNA HS Assay Kit (Invitrogen, ThermoFisher Scientific, Carlsbad, CA, USA).

For strains marked with HPI, adapter-compatible DNA was prepared using Ion Xpress™ Plus Fragment Library Kit and enzymatically fragmented for 5-12 minutes, resulting in a median fragment size of 350-450 bp and the libraries were prepared using the Ion Plus Fragment Library Kit. The resulting 400 bp insert libraries were used to prepare enriched, template-positive Ion PGM™ Hi-Q™ View Ion Sphere Particles (ISPs) with the Ion OneTouch™ 2 System. 850-flows sequencing was performed using the Ion PGM™ Hi-Q™ View Sequencing Kit with the Ion 318™ Chip Kit v2.

For genomes marked with UBa, genomic DNA was quantified using a NanoDrop spectrophotometer, as well as the Quant-iT DNA Assay Kit (Life Technologies, Paisley, UK) before sequencing. High-throughput genome sequencing was performed using a HiSeq 2500 machine (Illumina, San Diego, CA, USA).

Genomic DNA from strains marked with OiU was extracted using DNeasy Blood & Tissue kit (QIAGEN, Hilden, Germany). DNA concentration was measured using QuantusTM Fluorometer (Promega). High-throughput genome sequencing was performed either on Hiseq 2000 (2 × 100 or 2 × 150 paired-end reads) or Miseq (2 × 300 paired-end reads) sequencer (Illumina, San Diego, CA) following the manufacturer 's instruction.

Primary bioinformatics analysis

For the KI genomes, the raw sequencing reads were quality trimmed and filtered using TrimGalore! (http://www.bioinformatics.babraham.ac.uk/projects/trim_galore/) applying a minimum q-score of 30, and *de novo* assembled using SPAdes [10] with the –careful option. Contigs with very low coverage and that were shorter than 500 bp were discarded prior to annotation.

For the MHH genomes quality filtering was done with Trimmomatic version 0.36 [11] and the assemblies were performed with SPAdes genome assembler v. 3.9.0 and resulted in number of all contigs from 26 up to 117. Assemblies were quality controlled with QUAST[12].

For the HPI genomes, quality control of raw sequencing reads was performed using FASTQC (https://www.bioinformatics.babraham.ac.uk/projects/fastqc). Unbiased *de novo* assembly was performed using SPAdes genome assembler v. 3.5.0 in default mode and contigs shorter than 300 bp were removed.

For the UBa genomes, raw sequencing reads were quality trimmed and filtered using Trimmomatic v. 0.33 and the 100 bp short read paired-end data was assembled using the *de novo* assembly algorithm Velvet version 1.2.08[13]. The VelvetOptimiser script (v. 2.2.4) was run for all odd k-mer values from 21 to 99. The minimum output contig size was set to 200 bp with default settings, and the scaffolding option was disabled.

For the OiU genomes, Trimmomatic v. 0.35 was used to remove adapter sequences and low-quality bases from raw short reads data. Trimmed reads were then de novo assembled to produce contigs using SPAdes genome assembler v. 3.12.0 with the -careful option to reduce mismatches in the assembly. The minimum contig length was set to 200 bp.

Supplementary References:

1 Aro, P. *et al.* Peptic ulcer disease in a general adult population: the Kalixanda study: a random population-based study. *American journal of epidemiology* **163**, 1025-1034 (2006). https://doi.org:10.1093/aje/kwj129

2 Storskrubb, T. *et al.* A negative Helicobacter pylori serology test is more reliable for exclusion of premalignant gastric conditions than a negative test for current H. pylori infection: a report on histology and H. pylori detection in the general adult population. *Scandinavian journal of gastroenterology* **40**, 302-311 (2005). https://doi.org:10.1080/00365520410010625

3 Berthenet, E. *et al.* A GWAS on Helicobacter pylori strains points to genetic variants associated with gastric cancer risk. *BMC Biol* **16**, 84 (2018). https://doi.org:10.1186/s12915-018-0550-3

4 Vale, F. F. *et al.* Genomic structure and insertion sites of Helicobacter pylori prophages from various geographical origins. *Scientific reports* **7**, 42471 (2017). https://doi.org:10.1038/srep42471

5 Boltin, D. *et al.* Trends in secondary antibiotic resistance of Helicobacter pylori from 2007 to 2014: has the tide turned? *Journal of clinical microbiology* **53**, 522-527 (2015). https://doi.org:10.1128/JCM.03001-14

6 Latifi-Navid, S. *et al.* Ethnic and geographic differentiation of Helicobacter pylori within Iran. *PLoS One* **5**, e9645 (2010). https://doi.org:10.1371/journal.pone.0009645

7 Nell, S. *et al.* Recent acquisition of Helicobacter pylori by Baka pygmies. *PLoS genetics* **9**, e1003775 (2013). https://doi.org:10.1371/journal.pgen.1003775

8 Linz, B. *et al.* An African origin for the intimate association between humans and Helicobacter pylori. *Nature* **445**, 915-918 (2007). https://doi.org:10.1038/nature05562

9 Aftab, H. *et al.* Two populations of less-virulent Helicobacter pylori genotypes in Bangladesh. *PLoS One* **12**, e0182947 (2017). https://doi.org:10.1371/journal.pone.0182947

10 Bankevich, A. *et al.* SPAdes: a new genome assembly algorithm and its applications to single-cell sequencing. *Journal of computational biology : a journal of computational molecular cell biology* **19**, 455-477 (2012). https://doi.org:10.1089/cmb.2012.0021

11 Bolger, A. M., Lohse, M. & Usadel, B. Trimmomatic: a flexible trimmer for Illumina sequence data. *Bioinformatics* **30**, 2114-2120 (2014). https://doi.org:10.1093/bioinformatics/btu170

12 Gurevich, A., Saveliev, V., Vyahhi, N. & Tesler, G. QUAST: quality assessment tool for genome assemblies. *Bioinformatics* **29**, 1072-1075 (2013). https://doi.org:10.1093/bioinformatics/btt086

13 Zerbino, D. R. & Birney, E. Velvet: algorithms for de novo short read assembly using de Bruijn graphs. *Genome Res* **18**, 821-829 (2008). https://doi.org:gr.074492.107 [pii]
10.1101/gr.074492.107
