# Supplementary material for: Repeated out-of-Africa expansions of Helicobacter pylori driven by replacement of deleterious mutations
Source: Nat Commun. 2022 Nov 11;13:6842. doi: 10.1038/s41467-022-34475-3 (PMC9652371; doi:10.1038/s41467-022-34475-3)
Supplement: Supplementary file 2 — Description of Additional Supplementary Files [file 41467_2022_34475_MOESM2_ESM.pdf]

Description of Additional Supplementary Files

File Name: Supplementary Data 1
Description: Newly sequenced strains in this study

File Name: Supplementary Data 2
Description: Publicly available strains used in the study

File Name: Supplementary Data 3
Description: Summary of isolates sequenced in this study

File Name: Supplementary Data 4
Description: Assembly and Annotation statistics Newly Sequenced genomes

File Name: Supplementary Data 5
Description: All strains in the dataset and how they were used in the different analyses

File Name: Supplementary Data 6
Description: donors used for D-statistics calculations

File Name: Supplementary Data 7
Description: donors used for D-statistics calculations

File Name: Supplementary Data 8
Description: gene-by-gene African ancestry
