# Supplementary material for: Repeated out-of-Africa expansions of Helicobacter pylori driven by replacement of deleterious mutations
Source: Nat Commun. 2022 Nov 11;13:6842. doi: 10.1038/s41467-022-34475-3 (PMC9652371; doi:10.1038/s41467-022-34475-3)
Supplement: Supplementary file 4 — Reporting Summary [file 41467_2022_34475_MOESM4_ESM.pdf]

nature research

Corresponding author(s): Daniel Falush

Last updated by author(s): Oct 14, 2022

Reporting Summary

Nature Research wishes to improve the reproducibility of the work that we publish. This form provides structure for consistency and transparency in reporting. For further information on Nature Research policies, see Authors & Referees and the Editorial Policy Checklist .

Statistics

For all statistical analyses, confirm that the following items are present in the figure legend, table legend, main text, or Methods section.

n/a	Confirmed	
☐	☒	The exact sample size (*n*) for each experimental group/condition, given as a discrete number and unit of measurement
☐	☒	A statement on whether measurements were taken from distinct samples or whether the same sample was measured repeatedly
☒	☐	The statistical test(s) used AND whether they are one- or two-sided<br>*Only common tests should be described solely by name; describe more complex techniques in the Methods section.*
☐	☒	A description of all covariates tested
☐	☒	A description of any assumptions or corrections, such as tests of normality and adjustment for multiple comparisons
☐	☒	A full description of the statistical parameters including central tendency (e.g. means) or other basic estimates (e.g. regression coefficient) AND variation (e.g. standard deviation) or associated estimates of uncertainty (e.g. confidence intervals)
☒	☐	For null hypothesis testing, the test statistic (e.g. *F*, *t*, *r*) with confidence intervals, effect sizes, degrees of freedom and *P* value noted<br>*Give P values as exact values whenever suitable.*
☒	☐	For Bayesian analysis, information on the choice of priors and Markov chain Monte Carlo settings
☒	☐	For hierarchical and complex designs, identification of the appropriate level for tests and full reporting of outcomes
☒	☐	Estimates of effect sizes (e.g. Cohen's *d*, Pearson's *r*), indicating how they were calculated

Our web collection on statistics for biologists contains articles on many of the points above.

Software and code

Policy information about availability of computer code

Data collection

the simulation data was generated using a custom script run in SLiM v. 3.6 (https://github.com/EliseTourrette/Hpylori/tree/main/HpEurope)

Data analysis

For newly sequenced genomes
genome quality filtering:
KI genomes: TrimGalore!
MHH genomes: Trimmomatic v0.36
HPI genomes: FASTQC
UBa genomes: Trimmomatic v0.33
OiU genomes: Trimmomatic v0.35

assemblies:
KI genomes: SPAdes
MHH genomes: SPAdes v3.9.0, quality control with QUAST
HPI genomes: SPAdes v3.5.0
UBa genomes: Velvet v1.2.08
OiU genomes: SPAdes v3.12.0

For all genomes:
annotation:
prokka v1.12
genome size and contig/scaffold number collected from prokka output with MultiQC

alignment:
Snippy v3.2-dev

population structure:
fineSTRUCTURE v0.02
ChromoPainter v0.04

PCA:
PLINK v1.9

D-statistics:
popstats (no version)

chromosome painting:
ChromoPainterV2

dN/dS calculation:
PAML v4.7

Admixture graphs:
Treemix v1.12

Rate of non-adaptive non-synonymous amino acid substitutions:
GRAPES v1.1.1

R packages:
mvoutlier (function aq.plot())
PopGenome
Tidyverse
ggplot2

the scripts used for the analysis of the simulations, using R v4.1.1 and python v3.9.6, can be found here https://github.com/EliseTourrette/Hpylori/tree/main/HpEurope

For manuscripts utilizing custom algorithms or software that are central to the research but not yet described in published literature, software must be made available to editors/reviewers. We strongly encourage code deposition in a community repository (e.g. GitHub). See the Nature Research guidelines for submitting code & software for further information.

Data

Policy information about availability of data

All manuscripts must include a data availability statement. This statement should provide the following information, where applicable:

- Accession codes, unique identifiers, or web links for publicly available datasets
- A list of figures that have associated raw data
- A description of any restrictions on data availability

All newly sequenced genomes can be found in GenBank (https://www.ncbi.nlm.nih.gov/genbank/) under BioProject PRJNA479414. Individual accessions of the genomes can be found in Supplementary data 1. For the publicly available genomes, GenBank accessions or equivalents in other databases can be found in Supplementary Data 2. The entire dataset of 716 genomes have been deposited in Data Dryad, accessible here: https://doi.org/10.5061/dryad.v9s4mw70c.

Field-specific reporting

Please select the one below that is the best fit for your research. If you are not sure, read the appropriate sections before making your selection.

☐ Life sciences ☐ Behavioural & social sciences ☒ Ecological, evolutionary & environmental sciences

For a reference copy of the document with all sections, see nature.com/documents/nr-reporting-summary-flat.pdf

Ecological, evolutionary & environmental sciences study design

All studies must disclose on these points even when the disclosure is negative.

Study description	This study looks at the evolutionary history of a dataset of 716 Helicobacter pylori sequences sampled in different parts of the world.
Research sample	A dataset of 716 Helicobacter pylori whole-genome sequences was assembled, consisting of 213 newly sequenced isolates from Europe, Asia and Africa (Table Supplementary Data 1) and selected publicly available genomes (Table Supplementary Data 2). To complement the publicly available data, we included isolate collections from the following three main geographical areas: Europe, Middle East, North East Africa and South East Asia. This new dataset is summarized in (Supplementary Data 3), including citations and details on ethical approval for the respective cohorts. The European and Middle Eastern genomes were included to obtain a more

comprehensive mapping of ancestries within the area, the genomes from Central and North East Africa to have solid whole genome representation of the "Ancestral Europe" population. Lastly, the South East Asian genomes were chosen due to their unadmixed hpAsia2 background to serve as donors for hpAsia2 ancestry. For details on sample collection and bacterial isolation in the different cohorts, see Supplementary Methods

Sampling strategy	The cohorts, sampling procedure and bacterial isolation is detailed in the Supplementary Methods section together with the procedures for DNA extraction, library preparation, sequencing and primary bioinformatics
Data collection	Sequences were sequenced in five different centres: Karolinska Institute (Sweden), Hannover Medical School (Germany), Hellenic Pasteur Institute (Greece), Oita University (Japan) and the University of Bath (UK). The primary bioinformatics analysis (trimming, filtering, quality check and assemblies) were also done separately.
Timing and spatial scale	The clinical samples were collected in several different cohorts over the last 30 years and were selected to represent geographical areas or human populations rather than reflecting a specific time interval. They are not necessarily a representative sample either geographically or pathologically since H. pylori requires endoscopy, an invasive medical intervention and therefore are collected opportunistically, normally from middle age people with some kind of gastric complaint. The details on collection year, where available, are now added to Supplementary Data 2.
Data exclusions	Which genomes that have been used for what purposes and, if they have been excluded from some of the analyses, why that is, is detailed in Supplementary Data 5. We have also provided a statement pointing to this information in the Methods section.
Reproducibility	Multiple isolates from the same geographical zone/population were sampled in order to assess the variability of the different measurements.
Randomization	The group allocations, apart from geographical origin, were the H. pylori subpopulations, which were inferred from the analysis results (fineSTRUCTURE analysis, Supp. Figure 1.)
Blinding	As the categories in terms of population assignment were central to the downstream analyses and result interpretation, blinding of the group allocation was not suitable for this study.

Did the study involve field work? ☐ Yes ☒ No

Reporting for specific materials, systems and methods

We require information from authors about some types of materials, experimental systems and methods used in many studies. Here, indicate whether each material, system or method listed is relevant to your study. If you are not sure if a list item applies to your research, read the appropriate section before selecting a response.

Materials & experimental systems

n/a	Involved in the study	
☒	☐	Antibodies
☒	☐	Eukaryotic cell lines
☒	☐	Palaeontology
☒	☐	Animals and other organisms
☐	☒	Human research participants
☒	☐	Clinical data

Methods

n/a	Involved in the study	
☒	☐	ChIP-seq
☒	☐	Flow cytometry
☒	☐	MRI-based neuroimaging

Human research participants

Policy information about studies involving human research participants

Population characteristics	The clinical samples were collected in several different cohorts over the last 30 years and were selected to represent geographical areas or human populations. They are not necessarily a representative sample either geographically or pathologically since H. pylori requires endoscopy, an invasive medical intervention and therefore are collected opportunistically, normally from middle age people with some kind of gastric complaint.
Recruitment	Since H. pylori requires endoscopy, an invasive medical intervention, the samples were collected opportunistically, normally from middle age people with some kind of gastric complaint.
Ethics oversight	Ethical permission for the collection of human gastric biopsy material had been obtained for all cohorts, including informed consent from the participating individuals. For details on the board/committee and institution that approved each study protocol, see Supplementary Data 3:<br>Umeå University, Sweden<br>Nottingham University Hospitals NHS Trust and Swansea Bay University Health Board, UK.<br>Health Ethics Commission from the National Institute of Health Dr Ricardo Jorge.<br>Alexandra General Hospital and General Hospital Athens "Evangelismos-Polykliniki', Greece.<br>Rabin Medical Center institutional Ethics Committee, Israel.<br>Digestive Diseases Research Center, Shariati Hospital, Tehran University of Medical Sciences, Iran.<br>0002/ERCC/CBNO2 from the Cameroon Bioethics Initiative<br>Federal Ministry of Health of Sudan<br>Ethics Committee of the Bangladesh Medical Research Council (BMRC) (Dhaka, Bangladesh), and the Oita University Faculty of Medicine, Japan<br>Human Research Ethnics Committee of Faculty of Medicine, Thammasat University (Pathum Thani, Thailand), and the Oita University Faculty of Medicine, Japan

Note that full information on the approval of the study protocol must also be provided in the manuscript.
